# Transcriptional control of visual neural circuit development by GS homeobox 1

**DOI:** 10.1371/journal.pgen.1011139

**Published:** 2024-04-26

**Authors:** Alexandra R. Schmidt, Haiden J. Placer, Ishmael M. Muhammad, Rebekah Shephard, Regina L. Patrick, Taylor Saurborn, Eric J. Horstick, Sadie A. Bergeron

**Affiliations:** 1 Department of Biology, West Virginia University, Morgantown, West Virgina, United States of America; 2 Department of Neuroscience, West Virginia University, Morgantown, West Virgina, United States of America; Fred Hutchinson Cancer Research Center, UNITED STATES

## Abstract

As essential components of gene expression networks, transcription factors regulate neural circuit assembly. The homeobox transcription factor encoding gene, *gs homeobox 1* (*gsx1*), is expressed in the developing visual system; however, no studies have examined its role in visual system formation. In zebrafish, retinal ganglion cell (RGC) axons that transmit visual information to the brain terminate in ten arborization fields (AFs) in the optic tectum (TeO), pretectum (Pr), and thalamus. Pretectal AFs (AF1-AF9) mediate distinct visual behaviors, yet we understand less about their development compared to AF10 in the TeO. Using *gsx1* zebrafish mutants, immunohistochemistry, and transgenic lines, we observed that *gsx1* is required for vesicular glutamate transporter, *Tg(slc17a6b*:*DsRed)*, expression in the Pr, but not overall neuron number. *gsx1* mutants have normal eye morphology, yet they exhibit impaired visual ability during prey capture. RGC axon volume in the *gsx1* mutant Pr and TeO is reduced, and AF7 that is active during feeding is missing which is consistent with reduced hunting performance. Timed laser ablation of *Tg(slc17a6b*:*DsRed)*-positive cells reveals that they are necessary for AF7 formation. This work is the first to implicate *gsx1* in establishing cell identity and functional neural circuits in the visual system.

## Author summary

Visual ability depends on the proper assembly of neural circuits during development as orchestrated by a multitude of genetic and environmental factors. When visual system neurodevelopment is impaired, it can result in lifelong changes to an organism’s brain and behavior. *genomic screen homeobox 1* (*gsx1*) is expressed in developing visual systems of multiple organisms, yet its functional role remained underexplored. We found that *gsx1* is required for neuronal differentiation and neural circuit assembly in visual processing centers of the brain in zebrafish, the pretectum (Pr) and optic tectum (TeO). Retinal ganglion cell (RGC) axons connect the eyes to the brain in zebrafish by forming functionally distinct arborization fields (AFs) in these regions. We observed deficits in select AFs in zebrafish *gsx1* mutants including Pr AF7, AF8, and TeO AF10. AF7 function is linked to prey capture ability which is also impaired in *gsx1* zebrafish mutants. This work demonstrates for the first time in any vertebrate a novel and essential role for *gsx1* in functional development of the visual system. As a result, many testable RGC and other axon termination mechanisms can now be examined across all CNS areas that express *gsx1*.

## Introduction

The visual system eye-to-brain circuitry is a classic and extensively studied model to uncover neural circuit connectivity mechanisms that dictate innate and complex behaviors [1–3]. In vertebrates, visual information is transmitted to the brain via retinal ganglion cell (RGC) axons. Once RGC axons exit the eye, molecular cues including erythropoietin-producing hepatocellular (Eph) receptors and ephrin ligands [4,5] and Robo/Slit interactions [6–10] guide axons by promoting or inhibiting growth cone extension. These developmental instructions establish appropriate RGC axon pathfinding, synaptogenesis, and retinotopographic mapping, and many of these cues are conserved across mammalian and nonmammalian vertebrate visual systems [1,3,11–14]. Efforts to date have largely focused on identifying axonogenesis and synaptogenesis mechanisms in primary visual processing areas, and how RGCs target other critical brain regions remains incompletely understood [15–18]).

Zebrafish possess simplified visual circuitry that can be studied using chemical and tractable genetic tools. For example, zebrafish have monocular vision, and RGC axon connections occur only in the contralateral optic tectum (TeO) and pretectum (Pr) [19,20]. Due to their neuroanatomical simplicity and speed of anatomical and functional development, zebrafish were used in large-scale forward genetic screens to identify important genetic factors for visual system development [21–25]. These studies have furthered our understanding over the years of biological mechanisms underlying neurodevelopment and ocular function across vertebrates [26–29], yet like in many other systems, focused on the primary center for visual processing, the TeO.

Most RGC axons terminate in the zebrafish TeO, the neuroanatomical and functional equivalent structure of the mammalian superior colliculus (Sc). However, many of these RGC axons first terminate in the Pr before branching to send collaterals into the TeO [19,20]. Subclasses of RGCs have recently been defined based on their gene expression profiles in combination with their axon connectivity patterns [30,31]. Other studies have identified subclasses of RGCs based on dendritic morphologies in the retina, position in the retina, their axon projection patterns and positions in the TeO [20], their post-synaptic partners [10,31–33], and their activity patterns to definitive features of visual stimuli [30,34,35].

In zebrafish, RGC axons are further segregated into distinct retinorecipient neuropil regions called arborization fields (AFs) [19,20], that are analogous in many ways to known visual processing regions in mammals [36,37]. AFs are defined by localized high densities of presynaptic puncta, and they are numbered 1 to 10 based on their proximity away from the midline optic chiasm (OC) and progression along the optic tract [19,20]. AFs 1–9 terminate in the Pr and thalamus, and AF10 is in the TeO. Innate visual abilities have been differentially linked to distinct pretectal AFs, including computations underlying prey capture [38–42], reflexive eye movements [43,44], optic flow [45–49], changes in illumination [50], and visuomotor asymmetry in whole body turning [51]. These behaviors are mediated by select AFs 3 through 9, while AFs 1 and 2 have no defined functions in zebrafish to date [37]. AFs play key roles in driving visually-mediated behaviors, yet there still remains a gap in knowledge of the cellular and molecular mechanisms that contribute to initial formation and refinement of nine anatomically and functionally distinct pretectal AFs.

*genomic screen homeobox 1* (*gsx1*) encodes a homeobox transcription factor that is expressed in the developing hypothalamus, olfactory bulb, cerebellum, hindbrain, spinal cord, TeO, and Pr [52–55]. In mice, *gsx1* is expressed in the optic stalk at E11.5 [56] and in precursor cells that give rise to the Sc at E13.5 [52]. Integral roles for Gsx1 have been found in neural progenitor cell determination in ventral telencephalic regions in mice that ultimately give rise to cortical interneurons [57], and Gsx1 is known to limit proliferation and promote differentiation of glutamatergic and GABAergic interneurons in the mouse spinal cord [58]. In zebrafish, *gsx1* is co-expressed with markers of both excitatory and inhibitory neurons in dorsal spinal cord progenitor domains [55]. Examination of Gsx1 knockout (KO) mice have implicated Gsx1 in the hypothalamus and pituitary as contributing to normal hormone signaling that regulates body growth [59]. Similar roles in zebrafish have been identified in *gsx1* mutants [54], however, zebrafish *gsx1* mutants are adult viable and fertile while Gsx1 KO mice do not survive long past 3 weeks of age [59]. Gsx1-expressing neurons in the larval zebrafish brainstem modulate processing of acoustic stimuli in a behavioral testing paradigm related to sensory motor gating endophenotypes associated with neurodevelopmental disorders in humans [52,60]. While many roles for Gsx1 have been identified in early brain development and function across vertebrates, a role for Gsx1 in the developing visual system has not been explored despite its known expression there.

In this study, we examine neuronal differentiation in the visual system in *gsx1* zebrafish mutants and find that it is altered. We further characterize disrupted RGC axon connectivity patterns in the Pr and TeO, and we identify visually mediated behavioral deficits in *gsx1* mutants that are consistent with RGC axon termination defects in the Pr. Lastly, we test cellular mechanisms contributing to RGC axon termination in the Pr and how these are tied to prey capture behavior using localized laser ablation. Our findings show for the first time that *gsx1* is required for termination of a significant subset of RGC axons in the Pr and TeO and the visual abilities they mediate.

## Results

### *gsx1* is required for glutamatergic neuron differentiation in the pretectum but not neuron specification

**Fig 1:**
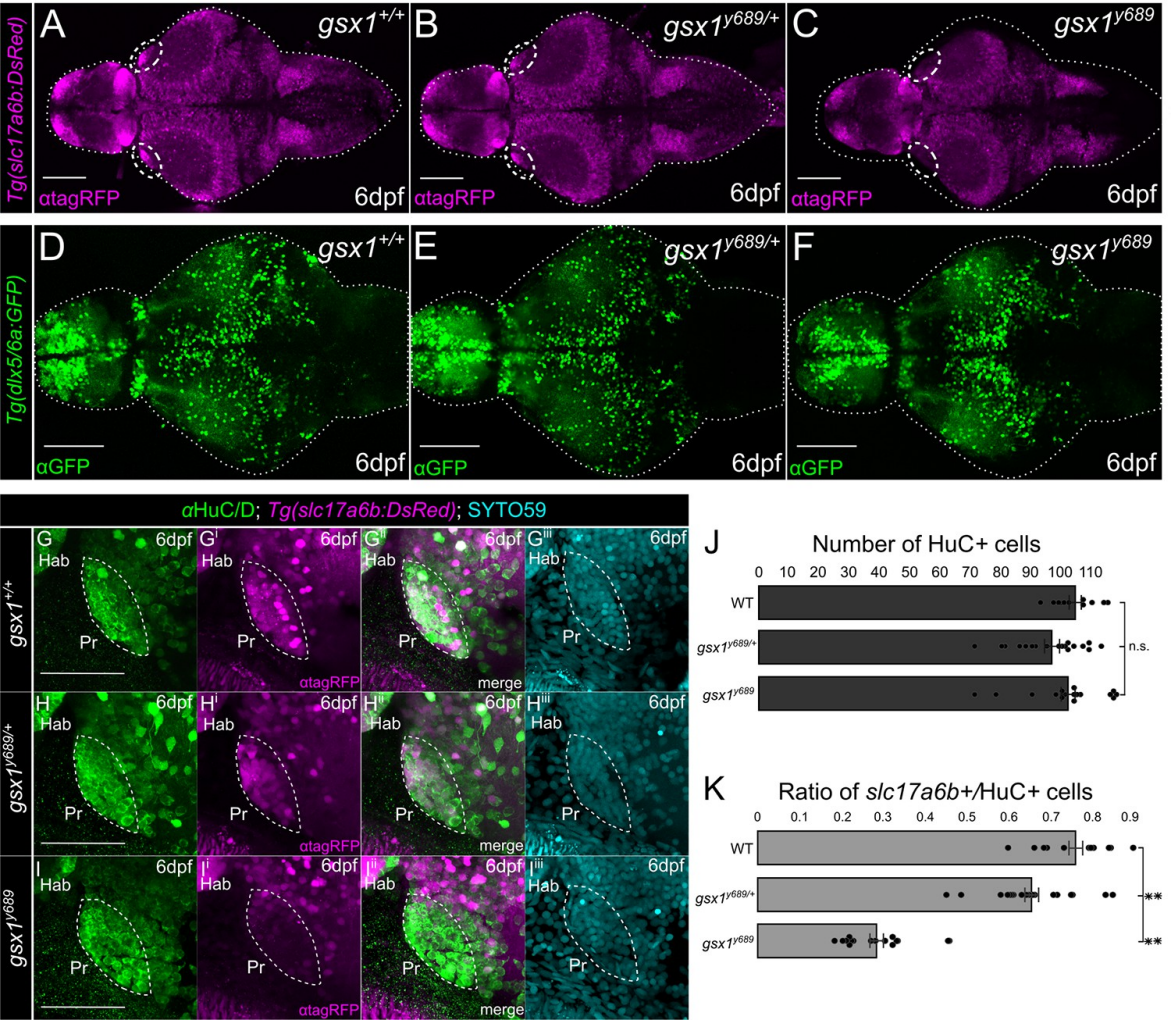
Examining excitatory and inhibitory neuron differentiation in *gsx1*^*y689*^. **(A-F)** Max projections of confocal z-stacks of *Tg(slc17a6b*:*DsRed)* and *Tg(dlx5a/6a*:*GFP)* at 6 dpf in *gsx1*^*+/+*^ (wildtypes), *gsx1*^y689/+^ (heterozygotes), and *gsx1*^*y689*^ (mutants). Scalebar = 100μm. White dashed oval **(A-C)** outlines pretectal region missing *slc17a6b* in *gsx1*^*y689*^. **(G-I**^**iii**^**)** Max projection of confocal z-stacks (~20μm) of pretectal region using *Tg(slc17a6b*:*DsRed)* (magenta) and *HuC/D* antibody (neurons, green) at 6 dpf. Cyan = SYTO59, nuclei. Scalebar = 50μm. **(J)** Bar graph with no significant differences found in total number of pretectal neurons by assessing *HuC/D* and SYTO59 staining across genotypes (single factor ANOVA F(2,43) = 2.05, p = 0.14). **(K)** Bar graph of average percentage of Pr *slc17a6b*-positive neuron counts out of *HuC/D* positive cell counts, *gsx1*^*+/+*^ (n = 11), *gsx1*^*y689/+*^ (n = 19), *gsx1*^*y689*^ (n = 16), F(2,43) = 3.21, *p* < .001. Post-hoc analysis of single factor ANOVA revealed *slc17a6b/HuC/D* ratio for *gsx1*^*+/+*^ (79.72 ± 2.64) is significantly different from *gsx1* ^*y689/+*^ (63.95 ± 2.92, *p*<0.001) and *gsx1*^*y689*^ (28.63±1.7, *p*<0.001). There is also a statistically significant difference between *slc17a6b/HuC/D* ratios in *gsx1*^*y689/+*^ and *gsx1*^*y689*^ (*p*<0.001). Power factor greater than 80% for all genotypes, *p*<0.05.

Due to previously identified roles for *gsx1* in the differentiation of glutamatergic neurons in the mouse and zebrafish spinal cord [55,58], we first investigated glutamatergic neurons in the zebrafish brain using a transgenic line reporting expression of the vesicular glutamate transporter, *solute carrier family 17 member 6b* (*slc17a6b*, formerly *vglut2a* and *vglut2*.*1*, line *Tg(slc17a6b*:*DsRed)*). These transgenic animals carried the *gsx1*^*y689*^ mutant allele that is an 11 base pair deletion in exon 1 of the *gsx1* gene resulting in a frame shift mutation encoding a premature stop codon before the Gsx1 DNA-binding homeodomain is encoded [54]. Slc17a6b has notable similarity to mammalian VGLUT2, belonging to a small group of proteins that mediate the uptake of glutamate by pre-synaptic vesicles [61–63], and *Tg(slc17a6b*:*DsRed)* is a well-established reporter line for glutamatergic neurons in zebrafish [64,65]. Examination of *Tg(slc17a6b*:*DsRed)* at 6 days post fertilization (dpf) revealed a robust reduction in expression in mutants (*gsx1*^*y689*^) in the anterolateral pretectal region of the visual system compared to wildtypes (*gsx1*^*+/+*^), and heterozygotes (*gsx1*^*y689/+*^) (Fig 1A–1C).

We also assessed changes in inhibitory neurons in the visual system using *Tg(dlx5a/6a*:*GFP)*, a transgenic line that reports expression of the transcription factor encoding genes that regulate inhibitory neuron differentiation in zebrafish [66,67] (Fig 1D–1F). We determined there was no obvious difference in expression of *Tg(dlx5a/6a*:*GFP)* in the *gsx1*^*y689*^ visual system; in fact, there were very few *Tg(dlx5a/6a*:*GFP)* positive cells in the anterolateral Pr region at all in wildtypes. Closer examination of this Pr region in *gsx1*^*y689*^ shows an apparent loss of inhibitory neuron projections coming into the Pr and expressing *Tg(dlx5a/6a*:*GFP)* (S1 Fig). We thus focused our continued efforts on quantifying *Tg(slc17a6b*:*DsRed)* expressing neuron changes further as this appeared to be the prominent resident neuron type that is affected in the Pr in *gsx1*^*y689*^.

We were able to assess if pretectal neurons were present in *gsx1*^*y689*^ using an antibody that recognizes a protein expressed in neurons, HuC/D [68] and a fluorescent dye that binds DNA to demarcate nuclei, SYTO59 [69]. Quantification revealed that *gsx1* mutants have the same number of Pr neurons within the region devoid of *Tg(slc17a6b*:*DsRed)* expression (Fig 1G–1J), however, in *gsx1*^*y689*^ only 28% of the neurons in that region express *Tg(slc17a6b*:*DsRed)*, while 66% of neurons were *Tg(slc17a6b*:*DsRed)*-positive in *gsx1*^*y689/+*^, and 76% of the total number of neurons were *Tg(slc17a6b*:*DsRed)*-positive in wildtypes (Fig 1K). These results show that *gsx1* determines the neurochemical identity of a large proportion of glutamatergic pretectal neurons, but not pretectal neuron number.

### RGC axon volume and trajectory analysis in *gsx1* mutants

**Fig 2:**
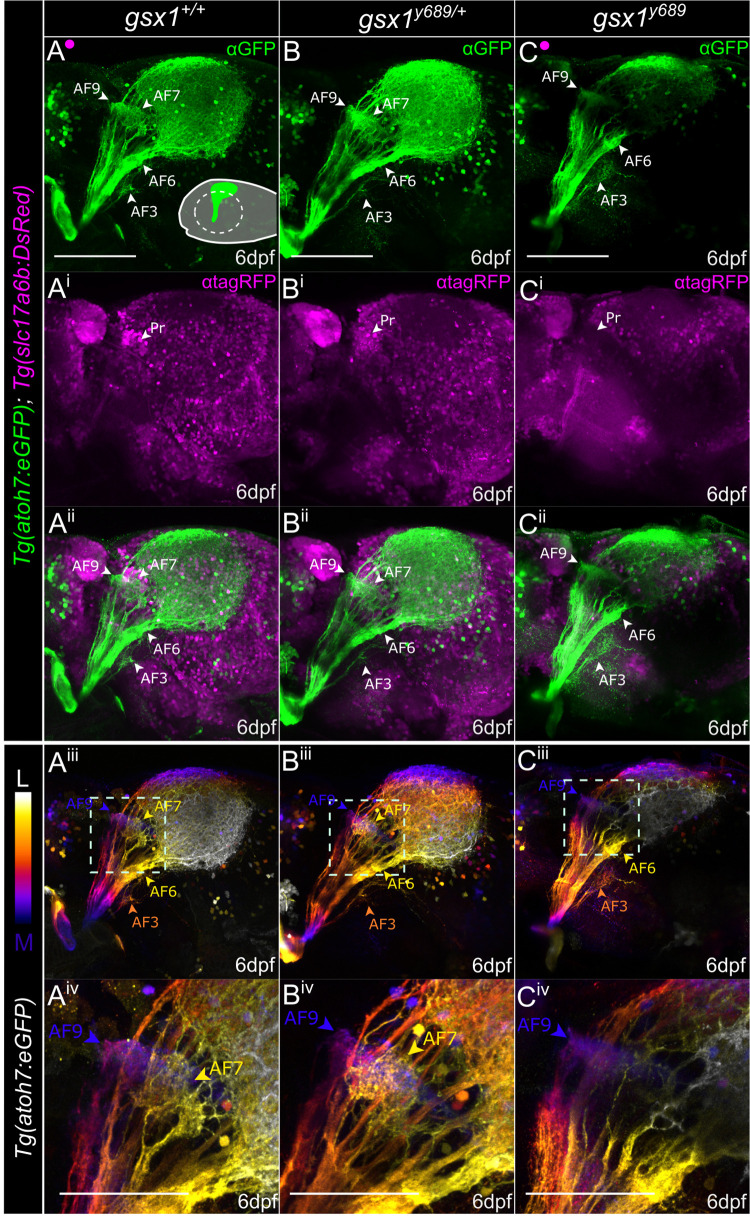
RGC axon termination is disrupted in *gsx1*^*y689*^. **(A-C)** Max projections of confocal z-stacks through the optic tectum from start of *Tg(atoh7*:*EGFP)* (green, RGC axons) labeling to end in **(A-A**^**ii**^**)** wildtypes (~85μm), **(B-B**^**ii**^**)** *gsx1*^*y689/+*^ (~82μm), and **(C-C**^**ii**^**)** *gsx1* mutants (~80μm). *Tg(slc17a6b*:*DsRed)* labels glutamatergic neurons (magenta), and white arrowhead, Pr, points to pretectal region lacking *Tg(slc17a6b*:*DsRed)* expression in *gsx1*^*y689*^. Schematic in **(A)** displays lateral orientation with dashed circle indicating removed eye. Scalebar = 100μm. **(A**^**iii**^**-A**^**iv**^**, B**^**iii**^**-B**^**iv**^**, C**^**iii**^**-C**^**iv**^**)** Depth coded view of same samples in A-C^ii^ to provide reference for some AF regions with color coordinated arrowheads for depth, AF9 = dark blue, AF7 = yellow, AF3 = orange, AF6 = yellow. **(A**^**iv**^**, B**^**iv**^**, C**^**iv**^**)** Zoomed in view of the pretectal AFs from dashed outline box in A^iii^, B^iii^, C^iii^. Scalebar = 50μm. AF9 can be seen in blue across each genotype, while AF7 is absent in *gsx1*^*y689*^ as seen by loss of yellow depth indicator.

Retinorecipient cells within the Pr and TeO cluster based on their unique gene expression profiles that do not appear to change with loss of retinal axon input beyond being delayed in timing [70]. These findings and others suggest that genetic profiles are hard-wired to exist in regions where RGC axons make connections, and they govern cellular mechanisms for the initial development and function of AFs [70–72]. Due to identified changes in neuronal identity in the *gsx1* mutant Pr (Fig 1), we looked to next assess the morphology of incoming RGC axons using *Tg(atoh7*:*eGFP)*, a transgenic line that demarcates RGCs and their axons with GFP expression [73]. From our labeling and imaging, we found that RGC axons in *gsx1*^*y689*^ fail to form AF7, AF8 may also be affected, and AF10 appears decreased in size, while other AFs including 3, 6, and 9 are still present at 6 dpf (Fig 2A–2C^ii^).

To better visualize individual AFs, we used depth color coding of Pr RGC axons from deepest (AF9) to most superficial, medial to lateral (Fig 2A^iii^–2C^iv^). We further examined yellow colored RGC axons, at the level where AF7 and AF8 are. In depth color-coded images for RGC axons, it appears AF9, which is known to develop near AF7, is unaffected across all genotypes. AF7 and AF8 are too close in proximity to resolve by depth color coding, thus we cannot reliably distinguish them. These findings show that with loss of *gsx1*, RGC axons do not form terminals in the region of *Tg(slc17a6b*:*DsRed)* reduction near AF7 and AF8 with additional defects present in RGC axon arbor size in AF10.

**Fig 3:**
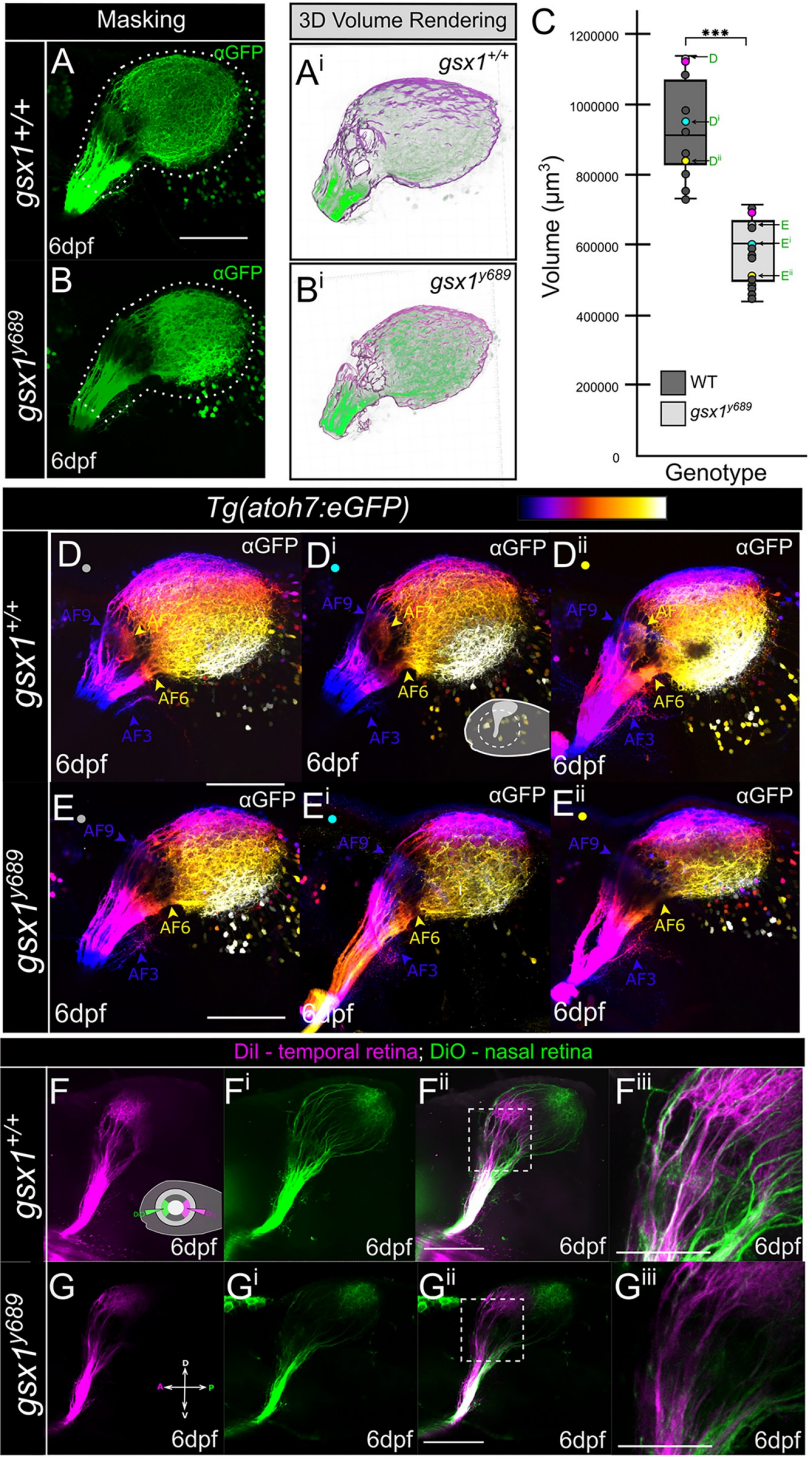
RGC axon volume and trajectory examination in *gsx1* mutants. **(A)** Example max projection of a confocal z-stack in wildtype and **(B)** *gsx1*^*y689*^, with *Tg(atoh7*:*eGFP)* anti-GFP labeling. Dashes around axons indicates regions of interest and mask application. **(A**^**i**^**-B**^**i**^**)** Example 3D volume rendering taken from Imaris when Labkit is used. **(C)** Box and whisker plot with individual data points included for volumes. There was a significant difference in volume of RGC axons between wildtype (M = 904100.00μm^3^, SD = 134984.32, SEM = 42685.79μm^3^, n = 10) and *gsx1* mutants (M = 564000.00μm^3^, SD = 80067.47, SEM = 24141.25μm^3^, n = 11); *t*(19) = 7.1, *p* < 0.001. Post-hoc power analysis = 100%. Colored dots correspond to Fig 2 images (magenta), and D-E^ii^ representative minimum (yellow), maximum (light gray), and average (cyan) axon volume images. **(D-D**^**ii**^**)** Wildtype and **(E-E**^**ii**^**)** mutant RGC axon images that have been depth color coded to show AF9 in blue as the deepest AF. These images represent the RGC axon phenotype variability in wildtypes and mutants as indicated in panel C. **(F-G**^**iii**^**)** Max projection of confocal z-stacks in wildtypes **(F-F**^**iii**^**)** and *gsx1*^*y689*^ **(G-G**^**iii**^**)**, with DiI (magenta) injected into the temporal retina and DiO (green) injected into the nasal retina. Scalebar = 100μm. **(F**^**iii**^ **and G**^**iii**^**)** Zoomed in pretectal region with consistent loss of AF7 in *gsx1*^*y689*^. Scalebar = 50μm.

Previous research shows that Gsx1 KO mice and *gsx1* zebrafish mutants have decreased body size [54,59]. Due to the observed reduction in RGC axon arbor size in *gsx1*^*y689*^ larval zebrafish at 6 dpf (Fig 2), we were interested in assessing if the 3D volume of RGC axons could be measured to confirm a size decrease despite overall changes in mutant body size. We used *Tg(atoh7*:*eGFP)* to label all RGC axons and examine lateral views of confocal z-stacks at 6 dpf in wildtype and *gsx1* mutants (Fig 3A, 3B^i^ and 3D–3E^ii^). At 6 dpf, the optic nerve extends from the midline to the contralateral side of the brain, and ventral AF1 and AF2 were not included in volume measurements. RGC axon volumes from AF3 to AF10 in *gsx1*^*y689*^ were found to be significantly smaller than wildtypes (Fig 3C). As significant body size deficits in zebrafish *gsx1* mutants have not been identified until 14 dpf [54], our findings support this axon phenotype at 6 dpf is likely irrespective of body size decrease in standard length.

Retinotopographic mapping in the zebrafish visual system has been examined by injection of lipophilic dyes into the retina in order to label specific subsets of anterior and posterior terminating RGC axons [20,21,74]. We predicted that due to the loss of RGC axon terminals in the more anterior AF7/8 region in *gsx1*^*y689*^ that they may terminate in off-target locations more posteriorly due to lack of receiving termination cues. To address this, DiI was injected into the temporal retina to distinctly label anterior RGC axons in the Pr and TeO, and DiO was injected into the nasal retina (Fig 3F and 3G^iii^). Consistent with prior results, loss of AF7/8 and AF10 size reduction were visually detected in *gsx1*^*y689*^. In six total samples, we did not observe anterior, DiI+ RGC axons from retinotemporal RGCs projecting into the posterior TeO. Overall, there is a reduced RGC axon volume in *gsx1*^*y689*^ Pr and TeO, and RGC axons maintain their general A/P retinotopographic termination zones.

### Prey capture and change in illumination behavior in *gsx1* mutants

**Fig 4:**
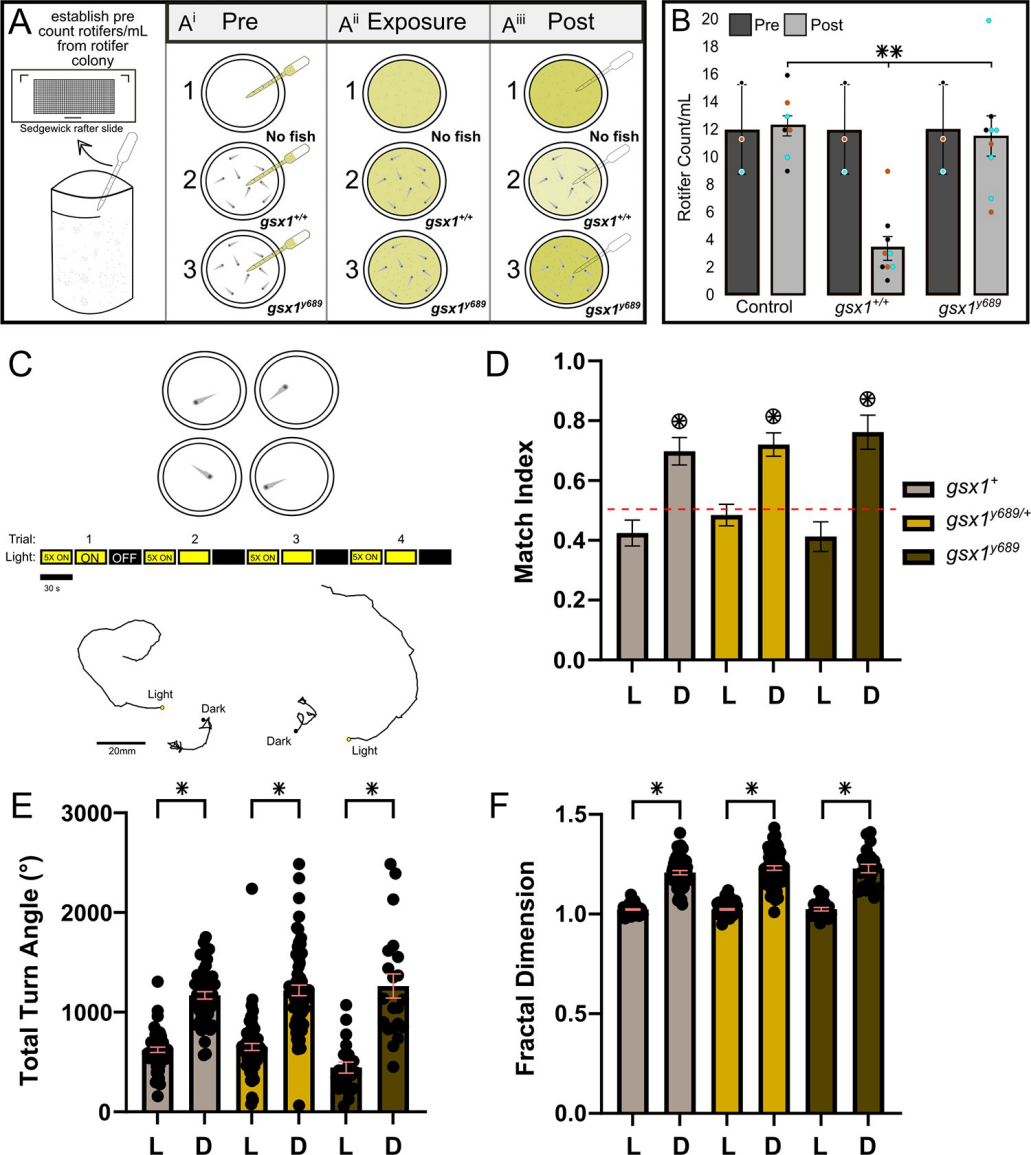
*gsx1* mutants have disrupted prey capture and normal light-mediated turn bias. **(A- A**^**iii**^**)** Experimental setup for prey capture assay; eight 7 dpf larvae per 6 cm dish, except in controls (no larvae). **(B)** Bar graph depicts rotifer/mL average across each group with individual data points showing all trials color coded (cyan, black, and orange) (n = 3) for controls (n = 7 dishes), wildtypes (n = 9 dishes), and *gsx1*^*y689*^ (n = 9 dishes); pre-feeding (dark grey bars), post-feeding (light grey bars). Power analysis revealed 100% for each condition, *p*<0.05. *gsx1*^*+/+*^ post count of rotifers/mL were statistically different from controls and *gsx1*^*y689*^, (F(2,24) = [3.44], *p*<0.001). Error bars displayed as ±SEM. No significant differences found between control and *gsx1*^*y689*^ post-feeding count analysis, *p* = 0.63. **(C-F)** Motor features extracted from the same pool of behavior tested larvae (n = 53 *gsx1*^*+*^ light grey bars, n = 65 *gsx1*^*y689/+*^ yellow bars, n = 21 *gsx1*^*y689*^ dark grey bars. Data collected from four independent clutches. **(C)** Schematic of recording field of view for turn bias assay. Four individual larvae recorded simultaneously. Not to scale (top). Light series during recordings showing light on (yellow) and light off (black) intervals. Scale bar represents a 30 second time period (middle). Representative traces during light on and off periods (bottom). **(D)** Match index during light on and dark trials (noted on the y-axis). Circle asterisk denotes p < 0.05 using a one-way Wilcoxon Signed Rank test to 0.5 representing random turn bias (shown with dotted red line). **(E-F)** Total turn angle and fractal dimension, representing other dark response motor features. * shows T test between light and dark responses for each genotype. Black circles show individual larva datapoints. Error bars displayed as ±SEM.

Functional analysis of AF7 has revealed its unequivocal role in zebrafish prey capture behavior [38–42,75]. We aimed to functionally validate the observed defects in AF7 in *gsx1*^*y689*^ by performing a prey capture assay in mutants compared to their wildtype cousins. We first determined an optimal number of live rotifers to the number of larvae per testing dish. To do this, we measured the number of rotifers per mL before adding larvae and after 2 hours of feeding (Fig 4A–4A^iii^). We then performed *in vitro* fertilization to obtain *gsx1* mutants and their wildtype cousins to assess prey capture at 7 dpf. *gsx1*^*y689*^ had higher post-feeding counts of rotifers within their environment compared to their wildtype cousins (Fig 4B), indicating an overall reduction in prey capture success. These results functionally confirm our neuroanatomical findings that AF7 is absent in *gsx1*^*y689*^, however, it does not rule out the possibility that defects in other AFs including AF10 could be contributing to this deficit as well.

To examine if visual behaviors attributed to different seemingly intact AFs are altered in *gsx1* mutants, we performed an analysis of motor turn bias. Following the loss of environmental illumination, larvae initiate a search pattern behavior characterized by increased same direction turning and increased local movement [76]. Over repeated trials, individual larva show a sustained turn direction preference or motor asymmetry (e.g. stable left or right turners), which is instructed by AF3 [51,77,78]. Wildtype, *gsx1*^*y689/+*^, and *gsx1*^*y689*^ larvae were placed individually into 10 cm diameter petri dish arenas and experienced four consecutive recordings of baseline illumination followed by loss of illumination, which elicits localized and persistent motor asymmetry behavior (Fig 4C). To assess motor asymmetry as an indicator of AF3 function, we used a match index (MI) where the turn direction of the last three dark trials is compared to the initial dark trial. Both heterozygotes and *gsx1* mutant larvae exhibited unidirectional turning comparable to wildtype controls (Kruskal-Wallis test, H = 0.314, p = 0.85) (Fig 4D). We also observed that wildtype, *gsx1*^*y689/+*^, and *gsx1*^*y689*^ zebrafish larvae displayed a significant increase in total turning (total turn angle, TTA) and localized movement (fractal dimension), suggesting overall normal photo-mediated response to the loss of full field illumination (Fig 4E–4F). These data show normal visually elicited directional turning (motor asymmetry) and implies that the ventral positioned AF3 is both morphologically and functionally intact in *gsx1*^*y689*^.

### *Tg(slc17a6b*:*DsRed)*-positive neurons are necessary for the termination of RGC axons in AF7

RGC axons follow complex sets of molecular cues upon eye exit, during guidance, and as they establish synapses on the contralateral side of the brain in the Pr and TeO [71]. Therefore, we aimed to determine if the deficit to AF7 in *gsx1*^*y689*^ is at the level of RGC axon termination or if additional disruptions exist before RGC axons reach the Pr. We found that *Tg(slc17a6b*:*DsRed)*-positive cells directly surround AF7 in wildtypes using *Tg(slc17a6b*:*DsRed)*;*Tg(atoh7*:*eGFP)* in combination with anti-Synaptotagmin (Znp1) antibody, a marker for presynaptic terminals (S2 Fig). We next confirmed the results of our previous gene expression studies [54] that *gsx1* is not expressed in the eye by performing RT-PCR on dissected eyes only or heads without eyes (S3A–S3A^ii^ Fig). We also examined the morphology of the retinal ganglion cell layer (GCL) at 6 dpf using *Tg(isl2b*:*GFP)* to label RGCs (S3B–S3D^ii^ Fig). RGCs within the eye appear qualitatively and quantitatively normal across *gsx1* genotypes (S3E Fig).

*gsx1* is known to be present in the Pr and TeO by 48 hpf and even earlier in neural precursor regions in the forebrain [54], consistent with the developmental period when RGC axons cross the forebrain midline [16]. We further examined optic nerve formation and optic chiasm crossing at 48 hpf, and no changes were detected across all genotypes (S4 Fig). These results align with previous findings that *gsx1*^*y689*^ does not have changes in early (1–2 dpf) forebrain patterning gene expression such as *dlx2a* and *dlx2b*, thus the cell and molecular substrate that RGC axons grow out upon is largely normal [54]. We hypothesized that the loss of AF7 is specifically related in some way to the loss of *Tg(slc17a6b*:*DsRed)* expression in *gsx1*^*y689*^ pretectal neurons.

**Fig 5:**
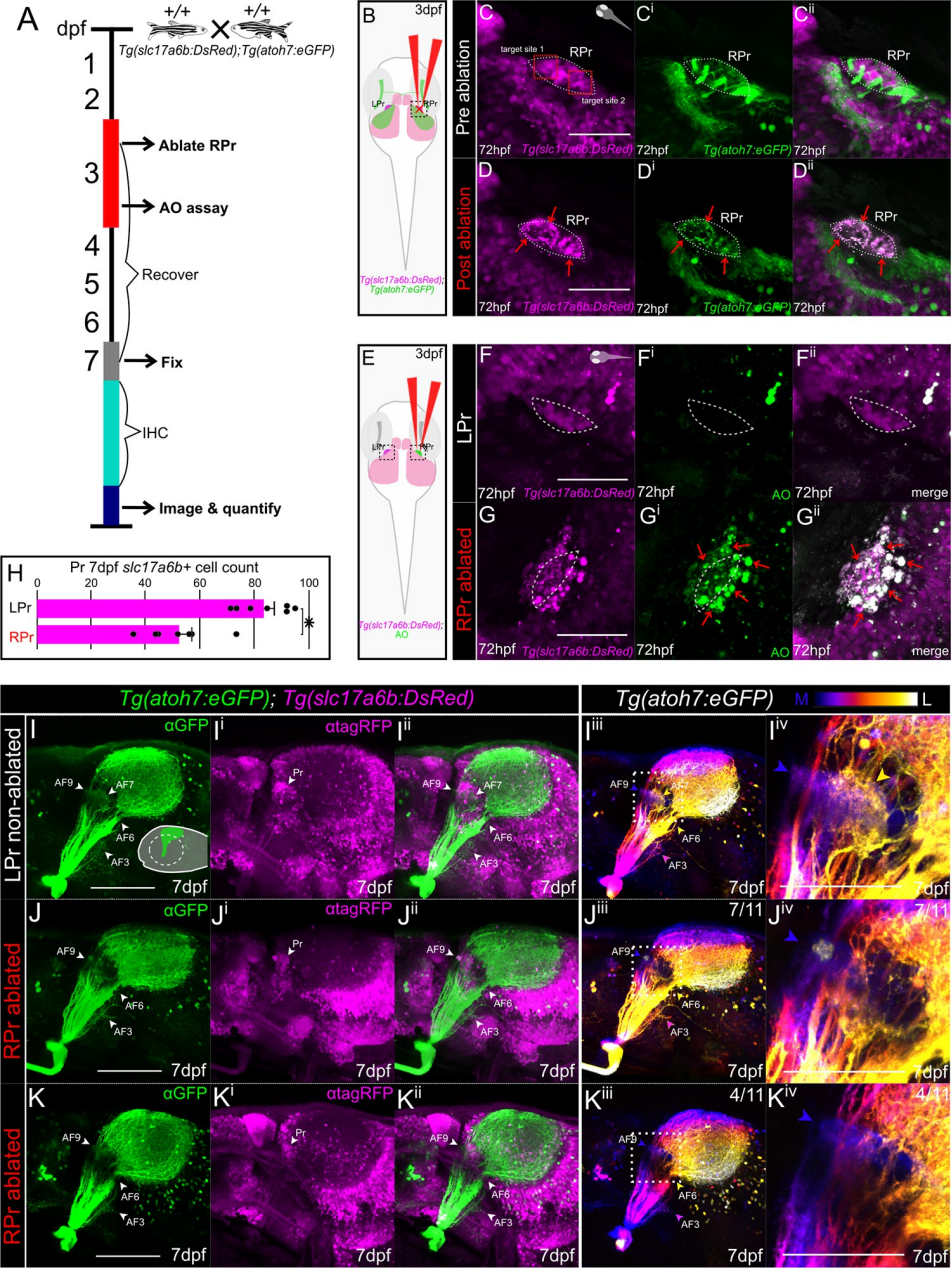
*Tg(slc17a6b)*-positive pretectal neurons are required for AF7 formation. **(A)** Experimental timeline: *gsx1*^*+/+*^;*Tg(slc17a6b*:*DsRed);Tg(atoh7*:*eGFP)* were raised until 72 hpf when they undergo unilateral ablation of the right pretectal (RPr) region. An acridine orange (AO) assay is performed on some samples at 72 hpf, hours post-ablation. Other ablated samples recover until 7 dpf. At 7 dpf samples are fixed and further undergo immunohistochemistry (IHC). **(B)** Schematic of unilateral RPr ablation in *gsx1*^*+/+*^;*Tg(slc17a6b*:*DsRed);Tg(atoh7*:*eGFP)* (magenta and green, respectively) at 72 hpf. LPr = left pretectum. **(C-C**^**ii**^**)** Pre-ablation of 72 hpf max projection of 2-photon z-stack through RPr separated by individual channels of *Tg(atoh7*:*eGFP)*, green, *Tg(slc17a6b*:*DsRed)*, magenta, and merged image channels. Two ablation sites are targeted and shown in C, red boxes. Dashed white outline indicates pretectal region. Orientation of sample is in right top corner. Scalebar = 50μm. **(D-D**^**ii**^**)** Post RPr ablation of same sample in C-C^ii^ at 72 hpf, red arrows indicate displacement of the fluorescent proteins. **(E)** Schematic of imaging planes in black dashed boxes after unilateral RPr ablation and then following AO staining. **(F-G**^**ii**^**)** AO staining compared to intact LPr in the same RPr ablated sample, using *Tg(slc17a6b*:*DsRed)* (magenta) while red arrowheads indicate increased acridine orange (green) in ablated side. **(H)** Bar graph of quantified *slc17a6b-positive* neurons at 7 dpf in the LPr and RPr following 72 hpf RPr ablation. The ablated RPr showed statistically significant decreases in *slc17a6b-positive* neurons compared to LPr side (n = 7), *t*(13) = 0.96, *p*<0.001. **(I-K**^**iv**^**)** Lateral 7 dpf max projections of confocal z-stacks of RGC axons in *Tg(atoh7*:*eGFP)* and *Tg(slc17a6b*:*DsRed)* and the same images depth color coded from medial (blue) to lateral (white), providing AF visualization, blue = AF9, yellow = AF7. Scalebar = 100μm. **(I-I**^**iv**^**)** Control (no ablation, n = 6), orientation schematic in right bottom corner. **(J-J**^**iv**^**)** 72 hpf unilateral RPr ablated resulting in partial AF7 disruption at 7 dpf (n = 7/11). **(K-K**^**iv**^**)** 72 hpf unilateral RPr ablated resulting in total AF7 disruption at 7 dpf (n = 4/11). **(I**^**iv**^**, J**^**iv**^**, K**^**iv**^**)** Zoomed in image from adjacent images with white dashed boxes for visualization of RGC axon AF patterns. Scalebar = 50μm.

In order to assess if *Tg(slc17a6b*:*DsRed)*-positive neurons in the Pr are involved in RGC axon termination and formation of AF7, we performed unilateral removal of pretectal *Tg(slc17a6b*:*DsRed)*-positive neurons at 72 hpf using 2-photon laser-ablation in *Tg(atoh7*:*eGFP)*;*Tg(slc17a6b*:*DsRed);gsx1*^*+/+*^ and examined the establishment of RGC axon terminals at 7 dpf (Fig 5A). The zebrafish visual system allows manipulations to be confined to one pretectal lobe, leaving the other side intact in the same animal for phenotypic comparison. At 72 hpf, the visual system is still developing, however, presumptive RGC axon neuropil regions can be seen, and the pretectal region of interest is morphologically distinct and identifiable for ablation using *Tg(slc17a6b*:*DsRed)* (Fig 5B–5D^ii^). Since RGC axons are near pretectal cells by 72 hpf, the possibility that they are also partly ablated upon *Tg(slc17a6b*:*DsRed)*-positive neuron ablation arises. However, studies have shown that the optic nerve can re-establish proper connections following transection by 4 days post injury (dpi) without additional RGC proliferation or cell death [79]. This finding establishes that RGC axons likely have the capability over the course of four days to find their proper position within the Pr if ablated. To examine this with our laser-ablation technique, we ablated a region of the optic nerve near AF6 using *Tg(atoh7*:*eGFP)* and examined RGC axon patterns 4 days post-ablation (S5 Fig). RGC axon patterns for AF6 are restored in ablated larvae at 7 dpf in comparison to controls by four days post-ablation (S5D and S5E Fig).

We confirmed cell death in our *Tg(slc17a6b*:*DsRed)*-positive neuron ablation technique through acridine orange (AO) staining on the ablated side of the Pr compared to the non-ablated side at 72 hpf, roughly four hours after ablation (Fig 5E–5G^ii^). Pretectal cell death on the ablated side was also seen at 6 dpf (3 days post ablation), however, AO staining is undetectable at 7 dpf (4 days post ablation), indicating that between 6 and 7dpf, the Pr cells may undergo cycling, consistent with findings from other studies examining these time points [80]. To understand how pretectal *Tg(slc17a6b*:*DsRed)*-positive neurons were impacted at 7 dpf, we quantified them on both the ablated and non-ablated Pr sides within the same samples, revealing a significant reduction in *Tg(slc17a6b*:*DsRed)*-positive neurons on the ablated pretectal side (Fig 5H).

RGC axon patterns at 7 dpf in unilateral ablated larvae show that AF7 formation was disrupted both partially (n = 7/11) and completely (n = 4/11) after pretectal *Tg(slc17a6b*:*DsRed)*-positive neuron ablation compared to non-ablated controls and the intact Pr side (Fig 5I–5K^ii^). The phenotypes for 7 dpf AF7 disruptions are specifically based on depth color coding (Fig 5I^iii^-5K^iv^). These findings support that pretectal *Tg(slc17a6b*:*DsRed)-*expressing cells are involved in RGC axon termination and formation of AF7, and ablated RGC axons at 72 hpf by our laser-focused methods have the capacity to reform connections over the course of four days if no other manipulations are performed to their recipient cells. While pretectal *Tg(slc17a6b*:*DsRed)* reduction post ablation is statistically significant, loss of *Tg(slc17a6b*:*DsRed)*-positive neurons does not completely match the reduction seen in *gsx1* mutants at 6 dpf (Fig 1H). However, it was sufficient to prevent AF7 from partly to completely forming, and it is also one full day after our initial quantification of *Tg(slc17a6b*:*DsRed)*-positive neurons in *gsx1*^*y689*^ when cell cycling and regeneration could be happening to some extent. We conclude that pretectal *Tg(slc17a6b*:*DsRed)-*expressing neurons determined by *gsx1* expression are required during development for RGC axons to terminate within the AF7/8 region.

### Retinotopographic mapping of RGC axons and prey capture behavior following the ablation of *Tg(slc17a6b*:*DsRed)*-positive neurons

**Fig 6:**
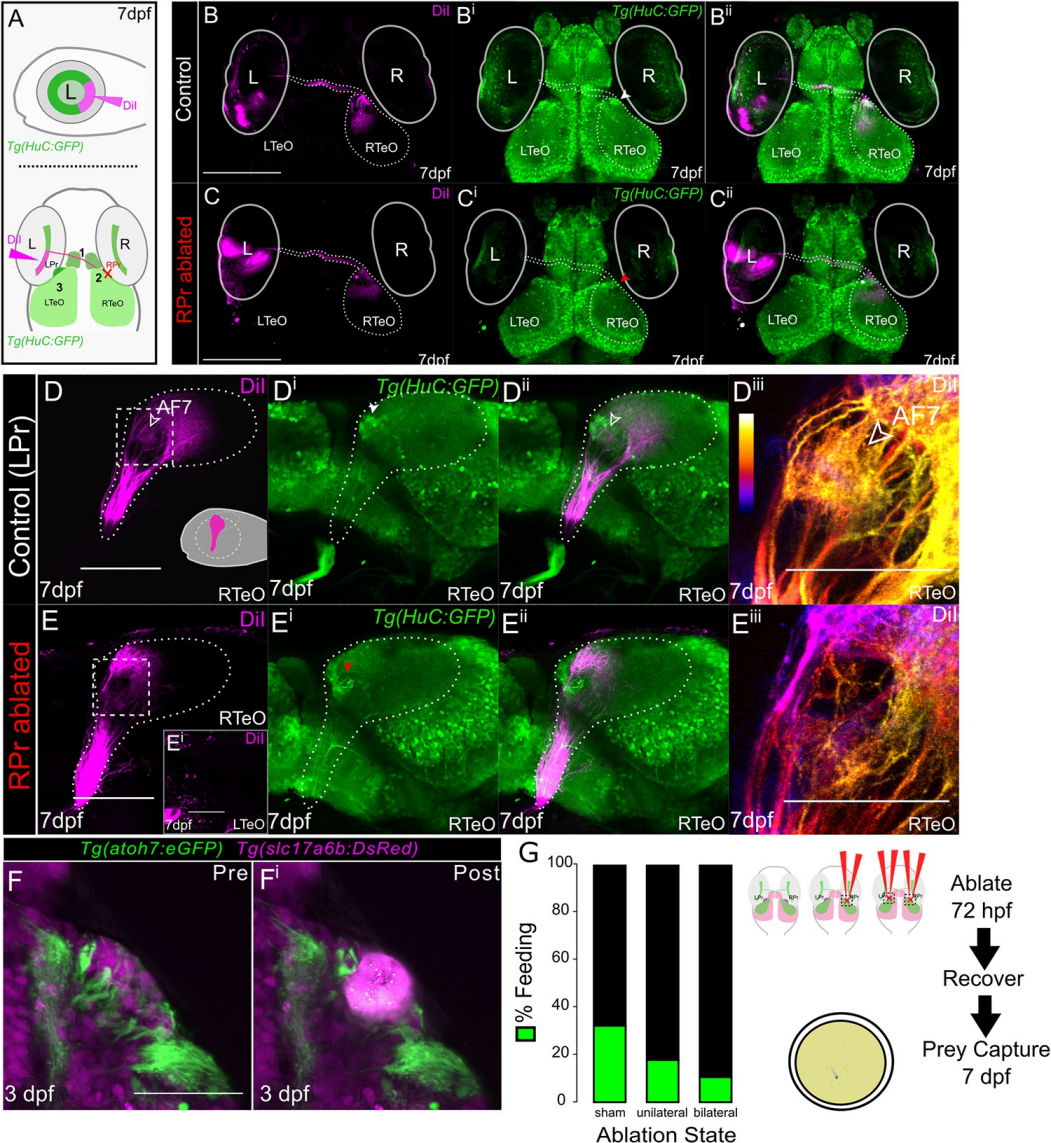
Retinotopographic order and prey capture defect is confirmed after pretectal ablations in the AF7 region. **(A)** Schematics indicating orientation and location of left eye (L) DiI injections and areas of RGC axon examination to the contralateral right TeO/Pr. Three black numbers indicate regions to examine labeling after ablation; 1) optic chiasm crossing, 2) anterior tectal labeling on contralateral lobe from injection, and 3) wandering DiI labeled RGC axons (left optic tectum = LTeO). R = right eye. **(B-B**^**ii**^**)** Max projections of 2-photon z-stacks (~200μm) in control non-ablated samples at 7 dpf (n = 10). DiI = magenta, green = *Tg(HuC/D*:*GFP)*. **(C-C**^**ii**^**)** Unilateral RPr ablations in *Tg(HuC/D*:*GFP)* wildtypes at 72 hpf, fixed at 7 dpf and DiI injected in temporal retina. Max projections of 2-photon z-stacks show proper optic chiasm crossing, anterior RGC axon arborization, and no RGC axons are seen targeting beyond the anterior contralateral tectum (n = 11). Red arrowhead indicates RPr that was ablated at 72 hpf. Scalebar = 200μm. **(D-D**^**iii**^**)** Max projection of confocal z-stacks of RTeO in lateral orientation showing DiI labeling in control (LPr) non-ablated samples (n = 5), (~85μm). Schematic of left eye DiI injection in D. White arrowhead indicates RPr region and red arrowhead indicates ablated RPr region. Scalebar = 100μm. **(E-E**^**iii**^**)** Lateral orientation with left eye removed of max projected confocal z-stacks of a RPr ablated sample and DiI labeling in RTeO (~85μm). **(D**^**iii**^**, E**^**iii**^**)** DiI image from D and E in white dashed box is depth color coded from medial (blue) to lateral (white) and zoomed in showing loss of AF7 (yellow). Scalebar = 50μm. (F-F^i^) Pre and post ablation images taken of the RPr in 72 hpf embryos used at 7 dpf for prey capture behavior. (H) After four days of recovery sham (n = 31), unilateral (n = 28), and bilateral (n = 28) ablated larvae were tested individually for prey capture behavior showing that sham ablated individuals ate significantly more often than bilaterally ablated individuals (sham to unilateral, x2(1) = 1.609, p = 0.2046; sham to bilateral, x2(1) = 3.975, p = 0.0462).

To further investigate RGC axon termination errors seen within our ablation experiments, we assessed retinotectal axon pathfinding following unilateral ablation of the pretectal cells. Ventrotemporal RGCs within the eye are known to project anteriorly including to AF7 and branch to form additional terminals within the TeO [20]. In order to assess if RGC axons occupy anatomically correct retinotectal termination zones following right-sided pretectal (RPr) cellular ablation using *Tg(HuC/D*:*GFP)* at 72 hpf, a lipophilic dye (DiI) was injected into the temporal region of the controlateral retina at 7 dpf (Fig 6A). Following ablation to the RPr, DiI-tracing via the left eye confirmed all RGC axons cross the optic chiasm and terminate within the anterior portion of the RPr and TeO consistent with control experiments at 7 dpf (Fig 6B–6C^ii^). We also observed that RGC axons do not show off-target or posterior projections within the Pr orTeO (Fig 6D–6E^ii^). Zoomed in images with depth color code for the RPr and TeO also indicate a phenocopy of *gsx1*^*y689*^, specific to AF7 following unilateral RPr ablations (Fig 6D^iii^ and 6E^iii^), as seen in previous experiments. Our ablation techniques using *Tg(HuC/D*:*GFP)* instead of *Tg(slc17a6b*:*DsRed)* produce similar phenotypes. These experiments show that RGC axons largely maintain proper retinotectal trajectories following the ablation of pretectal cells.

We next investigated whether *Tg(slc17a6b*:*DsRed)*-positive cell ablation inducing partial to complete loss of AF7 had any effect on prey capture behavior. *Tg(slc17a6b*:*DsRed)*-positive cell ablations were performed with a new digital laser control unit at 72 hpf either unilaterally or bilaterally in the pretectum region surrounding the AF7/8 area (Fig 6F–6F^i^). Control individuals were sham ablated and underwent all other procedures during rearing and experimental manipulation except for delivery of pulses of high intensity laser light. Larvae were tested individually for eating live rotifers in a 12-well plate over the course of two hours at 7 dpf following a four-day recovery period. 32.3% (n = 10/31) of sham ablated larvae consumed a significant number of rotifers above counting error (n > 2) while 17.9% (n = 5/28) and 10.7% (n = 3/28) of unilaterally and bilaterally ablated larvae respectively consumed rotifers. Bilateral ablation resulted in a significant decrease in feeding behavior compared to the sham ablation (Fig 6G) (x^2^(1) = 3.975, p = 0.0462), and unilateral ablation caused a feeding decrease that was not statistically significant. These results align with AF7 role in feeding behavior, and our RGC axon terminal loss in AF7 following pretectal cell ablation.

## Discussion

**Fig 7:**
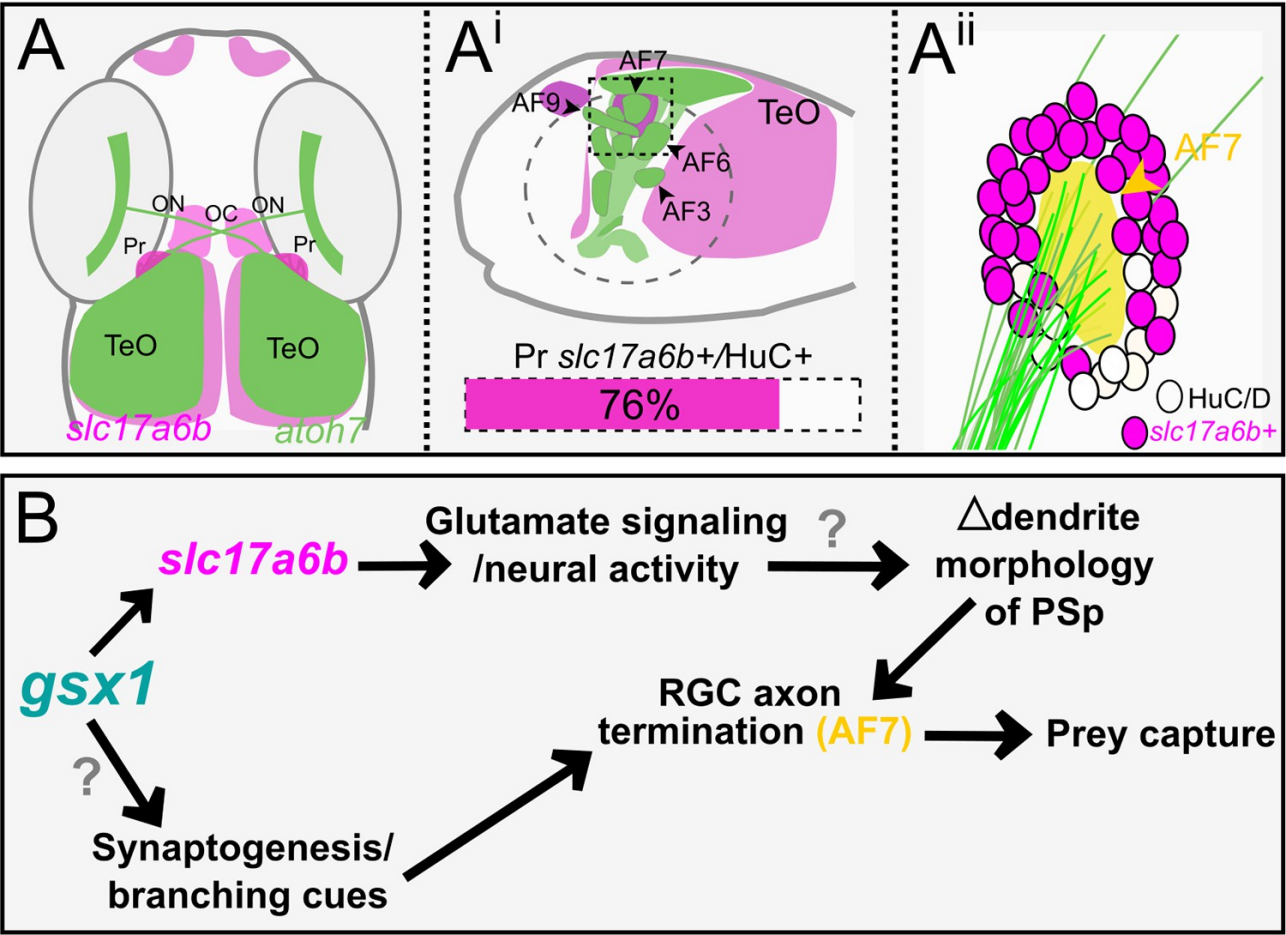
A model for *gsx1* function in the visual system. **(A)** Summary schematic of *Tg(slc17a6b*:*DsRed)* expression (magenta) and RGC axon patterning via *Tg(atoh7*:*eGFP)* expression (green) at 6 dpf in wildtypes (*gsx1*^*+/+*^). **(A**^**i**^**)** Lateral view of the TeO and pretectal arborization fields (AFs) (adapted from (20)) with *Tg(slc17a6b*:*DsRed)* expression (magenta). Wildtype pretectal *Tg(slc17a6b)/*HuC/D*+* cell ratio is shown with magenta bar. **(A**^**ii**^**)** Zoomed in schematic of RGC axons for AF7 surrounded by *Tg(slc17a6b*:*DsRed)-positive* cells from the outlined box in Ai. **(B)** Model of how *gsx1* contributes to pretectal neuronal identity and neural circuit assembly. Parvocellular superficial pretectal nucleus (PSp).

In this study, *gsx1* is shown for the first time to contribute to the differentiation of glutamatergic neurons in the visual system that aide in establishing proper eye to brain neural circuit connectivity during development. We found that in *gsx1* mutants, RGC axon termination is disrupted including the formation of AF7, possibly AF8, and AF10. In wildtype larvae, *Tg(slc17a6b*:*DsRed)*-expressing neurons directly surround AF7, and we establish through cellular ablation and RGC axon tracing techniques that the formation of AF7 is dependent on pretectal *Tg(slc17a6b*:*DsRed)*-positive neurons. We also confirm that *gsx1* is not expressed in the eye, and *gsx1* mutants have normal GCL morphology in the retina. We further examine RGC axon pathfinding in the forebrain and identify that *gsx1*^*y689*^ have typical patterns of RGC axon exit from the eye and normal optic chiasm formation. In *gsx1* mutants and following the removal of pretectal *Tg(slc17a6b*:*DsRed)* or *Tg(HuC/D*:*GFP)*-positive neurons during early development, we establish that RGC retinotectal axon anterior/posterior organization is maintained at larval stages. Additionally, we reveal that the visually mediated behavior of prey capture is decreased in *gsx1* mutants and following *Tg(slc17a6b*:*DsRed)*-positive neuron ablation, which is consistent with the functional profiles of AF7 and its absence in these mutants and in ablation experiments. In summary, pretectal neurons expressing *Tg(slc17a6b*:*DsRed)* are dependent on *gsx1* for differentiation, and these *Tg(slc17a6b*:*DsRed)*-positive neurons influence RGC axon termination at AF7 to drive visual behavior (Fig 7). Recent data based on functional and neuroanatomical findings suggests a great majority of the neurons that provide local (postsynaptic) communication to AF7, reside within the migrated pretectal region (M1) or superficial parvocellular pretectal (PSp) nucleus [36,37,81]), and some of these molecular cues could be part of the Gsx1-regulated gene expression network. All together we demonstrate the importance of *gsx1* for the development and function of visual neural circuits for the first time in any vertebrate.

### Pretectal *Tg(slc17a6b*:*DsRed)*-expressing neurons are involved in the development of AF7

Our findings are in line with identified roles for Gsx1 in the differentiation of neuronal subtypes in mouse and zebrafish [55,57,58]. Recent findings in mice indicate that the reintroduction of a lentivirus encoding Gsx1 can mediate functional recovery of neurons following spinal cord injury by increasing the number of glutamatergic and cholinergic neurons [82]. Our results indicate that with the loss of *gsx1*, mutants have changes to specific visual brain regions targeted by RGC axons. RGC axon termination errors are specific to AF7, AF8, and AF10, suggesting that the molecular identity of these recipient neurons is key to shaping incoming RGC axon synapses. Although *Tg(slc17a6b*:*DsRed)* is widely used to mark the majority of glutamatergic neurons in zebrafish, other vesicular glutamate transporters do exist within zebrafish, including *slc17a6a* (formally *vglut2b*), *slc17a7a* (formally *vglut1*) and *slc17a8* (formally *vglut3*) [65]. However, Slc17a6a is not uniquely detectable by antibody or transgenic line thus far and is reported to have overlapping expression profiles with that of *slc17a6b*. *slc17a7a* is also weakly expressed within the visual system, and *slc17a8* has been reported to have minimal expression, and therefore they were not included in our investigation. Despite its broad expression, the defects we observed in *Tg(slc17a6b*:*DsRed)* expression were very specific in only the regions where *gsx1* is also expressed in the the pretectum and extended into the brainstem and hypothalamus as well.

Global loss of normal *slc17a6b* function in the *blumenkohl* (*blu*) mutant results in reduced visual ability [65]. In addition, expanded RGC axon arbors with enlarged receptive fields are observed in the *blu* TeO [65]. This study confirmed that other glutamate transporters were still available but were not enough to overcome visual fatigue and expansion of RGC axon arbors with loss of *slc17a6b*, highlighting glutamatergic neurotransmission at the retinotectal synapse is mainly modulated through *slc17a6b*. Previous studies have also investigated retinotectal glutamate signaling in zebrafish through bath application of antagonists for the receptors N-methyl-D-aspartate (NMDA), α-amino-3-hydroxy-5-methyl-4-isoxazolepropionic acid (AMPA), and kainate (KA) [65,83], with resulting phenotypes consistent with *blu* and enlarged RGC axon arbors if blockage occurred at different times during visual system development. However, these experiments have examined RGC axon patterns following global changes to glutamate signaling, namely on both pre and postsynaptic sides of the terminal synapses. Our findings show that *Tg(slc17a6b*:*DsRed)* is decreased selectively in the Pr in *gsx1*^*y689*^ while the eye remains normal due to the absence of *gsx1* expression there. Often, RGC axons have been shown to be rapidly eliminated when unsuitable postsynaptic connections are not found [80,84], which may explain loss of AF7 without aberrant axon pathfinding. Our model is the first to implicate *slc17a6b* in the Pr alone as having a role in the development of AFs 7, possibly 8, and 10 requiring glutamatergic neurotransmission on the postsynaptic side of pretectal neural circuits to provide incoming RGC axons with cues for termination. Neurons that are less active during development may have less suitable dendrite morphology for normal synaptogenesis to occur, leading us to test this and other hypotheses about these *slc17a6b*-positive pretectal cells surrounding AF7 further as summarized in Fig 7B. It is also possible that transcriptional target genes of Gsx1 include known and novel axon termination, synaptogenesis, and/or branching cues for RGC axons in the Pr and TeO that are worth further investigation in light of this subtle but impactful phenotype.

### Visual neuropil region size is altered, but RGC axon topography is maintained

We discovered that the total RGC axon volume in *gsx1* mutants was reduced and can be easily visualized by quantifying three-dimensional axon volume from AF3 to AF10. Pretectal AFs terminate in anterior retinotectal regions and can be labeled with lipophilic dyes injected into the temporal retina [20]. We hypothesized that *slc17a6b* expressing pretectal neurons may produce stop signals for incoming RGC axons to terminate and upon loss of these signals, that these axons may occupy and target alternative regions. We analyzed retinotectal targeting and discovered that RGC axons do not expand across more posterior Pr and TeO or other locations despite AF deficits. AF7/8 and anterior TeO (AF10) designated axons may occupy unconventional anterior regions such as another pretectal AF, which would not be fully accounted for in this analysis. These subtle defects may be more easily detected using newly developed transgenic tools [30] to label where these axons ultimately reside in *gsx1*^*y689*^. It would be interesting to explore additional time points in development with more advanced genetic and imaging tools in hand to pinpoint when RGC axon volume differences are initiated in *gsx1*^*y689*^ and if critical windows dependent on *gsx1* shape morphological patterns during axon targeting and refinement of connections.

### Visually mediated dysfunction linked to neurodevelopmental changes

Prey capture requires neural circuits for visual perception, motor control, and decision making, which together give rise to the innate visually mediated response [85]. The existence and activity of AF7 have been highly linked to prey capture abilities, and this region within our studies was shown to be morphologically impacted with loss of *gsx1*. Both the left and right pretectal regions may function together during different phases of prey capture, and integration of interhemispheric tectal neuron populations serves to mediate motor coordination programs [41,86–88]. We suspect that there are other changes to be identified in neuronal differentiation in the TeO based on the documented expression of *gsx1* localized to the outer edges of the tectal neuropil [54], which likely further contribute to positional changes to incoming RGC axons based on the reduction of AF10 that we measured. AF10 reduction could also be due to axon branching failures upstream in the Pr region affected in *gsx1* mutants. These connections may play a role in sensorimotor processing and integration of information for behavioral decision making. As *gsx1* is also expressed in the hypothalamus which is another main driving center in the brain for feeding behavior, we must also consider the role that it plays there. However, these and other changes throughout the CNS related to loss of *gsx1* are difficult to dissect out given the initial deficit that we identified in AF7 without more advanced genetic tools currently in development stages. Nonetheless, disruption of AF7 alone in our ablation experiments proved to negatively impact prey capture behavior as in mutants which allows us to partly rule out effects on this behavior as being related to reduced AF10 arbor size. In addition, *gsx1* mutants exhibited normal turn bias behavior and have an intact and functional AF3. These results are in line with the idea that the pretectum is essential in relaying visual information to the TeO for further processing and behavioral action, and sensory input responses have been segregated to distinct pretectal AFs with limited crosstalk between these regions.

Since *gsx1*^*y689*^ are adult viable, they will make an excellent model to assess how early neurodevelopmental changes in the visual system influence later life behaviors that rely on intact visual ability such as social behaviors and learning. In addition, studies have shown that zebrafish without *gsx1*-expressing neurons and mouse Gsx1 KOs have disrupted pre-pulse inhibition (PPI) [52,60], a sensory gating behavior often disrupted in neurodevelopmental disorders (NDDs) such as schizophrenia [89]. Clinical populations of patients diagnosed with schizophrenia also have deficits related to the inhibition of saccadic eye movement reflexes, called anti-saccade errors [90]. Further exploration of a role for *gsx1* in the development of visually mediated behaviors such as examination of saccadic eye movements may elucidate additional functions for *gsx1* in sensory processing that are widely linked to NDDs [90,91].

Upon functional loss of *gsx1*, there are likely changes to known and novel molecular cues in the Pr and TeO influencing RGC axon termination, synaptogenesis, and branching. Our ablation techniques for assessing pretectal *Tg(slc17a6b*:*DsRed)*-positive cells early in development likely also disrupt these cues that guide RGC axons to their proper retinotopographic locations in a very localized way. Future studies to understand the full repertoire of molecular networks that Gsx1 regulates will uncover potentially conserved developmental mechanisms that influence RGC axon termination within visual neural circuits. Importantly, these studies link *gsx1* to sensory processing disruptions in the visual system in addition to the ones it has already been linked to in the auditory system. Across the CNS, *gsx1* appears to be a key player in sensory processing neural circuit development and function.

## Materials and methods

### Ethics statement

All aspects of this study were approved by the West Virginia University (WVU) Institutional Animal Care and Use Committee (IACUC), protocol number 1908027871. The WVU Office of Lab Animal Research (OLAR) offers guidance and support to zebrafish researchers at WVU, and WVU is AAALAC International accredited.

### Zebrafish husbandry

Adult zebrafish were maintained as described previously [54] at 25–28°C on a 14h/10h light/dark cycle. All embryos were staged under a dissecting scope [92,93] and raised in E3 embryo media (pH 7.4; 0.005M NaCl, 0.00017M KCl, 0.00033M CaCl, 0.00033M MgSO4.7H_2_0, 1.5 mM HEPES) at 28.5°C in an incubator with a 14h/10h light/dark cycle. Embryos and larvae used for histochemical techniques and ablations had E3 exchanged for 0.003% phenylthiourea (PTU) in E3 to prevent pigmentation. Wildtype (WT, *gsx1*^*+/+*^), *gsx1* heterozygotes (*gsx1*^*y689/+*^), and *gsx1* mutant (*gsx1*^*/y689*^) fish lines in a TL background [54], and transgenic *Tg(slc17a6b*:*DsRed)* [64], *Tg(dlx5a/6a*:*GFP)* [66,67], *Tg(atoh7*:*eGFP)* [73], *Tg(HuC/D*:*GFP)* [94], and *Tg(isl2b*:*GFP)* were used for these studies.

### Immunohistochemistry (IHC)

For histochemical techniques, *gsx1*^*y689/+*^ in various transgenic backgrounds were in-crossed to obtain offspring. Desired ages and verification of transgene expression patterns is obtained before fixation in 4% PFA in 1X phosphate buffered saline (PBS) at 4°C overnight. The following day, embryos and larvae are washed in 1X PBS, and undergo genotyping from dissected tail tissue, and brain and/or eye dissection is performed as needed before continuing with immunolabeling in order to achieve better penetration of antibodies. Repeating 1X phosphate TritonX (PTx) washes are completed at room temperature and then followed by 100% cold acetone treatment antigen retrieval at -20°C. Samples were then incubated in a blocking solution (5% normal goat serum, 1% DMSO in 1X PTx) for 1 hour at room temperature and then provided fresh block and incubation with primary antibodies (Table 1) at 4°C at a range of 2–4 overnights. Primary antibodies are then removed, and samples are washed with 1X PTx several times. Secondary antibodies are added (Table 1) with the same concentration of blocking reagents used previously, for 1–2 overnights at 4°C before additional washing in 1X PTx and storage in 1X PBS at 4°C just prior to sample mounting on slides and imaging.

**Table 1:** List of reagents used.

**Primers and Plasmids**						
Gene	Primer sequence			Used for		Additional Information
*gsx1* ^*y689*^	FW: 5’-TCCAGATCCACGACAGTTCC-3’ RV: 5’-TGACTGCTGCTATTTTCTGTTGA-3’			Genotyping		[54]
*gsx1*	FW: 5’-AGCATTTGGTACACGAGCGA-3’ RV: 5’-GGTGTGGCGTACAGAGTCTT-3’			Semi-quantitative RT-PCR		[54]
*ef1a*	FW: 5’-TACAAATGCGGTGGAATCGAC-3’ RV: 5’-TGTGCAGACTTTGTGACCTTG-3’					
**Antibodies and DNA stain**						
		Manufacturer	Item #		Host / Dilution	Reference
Primary						
αGFP		Thermo Fisher	NB1001614		Chicken / 1:800	-
αtagRFP		Thermo Fisher	NC0549849		Rabbit / 1:600	[52]
HuC/D		Thermo Fisher	A21271		Mouse / 1:500	[94]
SYTO59		Thermo Fisher	S11341		1:10,000	[69]
Secondary						
Goat α-mouse Alexa 488		Thermo Fisher	A11029		Mouse / 1: 1:800	-
Goat α-chicken Alexa 488		Thermo Fisher	A11039		Chicken / 1:1000	-
Goat α-mouse Alexa 633		Thermo Fisher	A21050		Mouse / 1:600	-
Goat α-rabbit Alexa 488		Thermo Fisher	A11008		Rabbit / 1:1000	-
Goat α-rabbit Alexa 568		Thermo Fisher	A11011		Rabbit / 1:1:800	-

### Behavior analyses

For group prey capture behavior, adult *gsx1*^*+/+*^ or their siblings, *gsx1*^*y689*^, underwent separate *in vitro* fertilization (IVF) rounds to acquire two groups of cousin larvae raised side-by-side for behavior testing. 12mL of E3 was exchanged daily (n = 30 individuals per 6 cm dish) until 7 dpf. At 7 dpf, a 100mL sample of the rotifer colony was acquired, filtered, and diluted in E3. A Sedgewick Rafter counting slide was used to assess how many rotifers were present per mL, establishing a pre-count that was recorded and added to individual dishes. Groups of n = 8 larvae were then transferred to each individual dish. One dish contained no larvae to account for multiplying rotifers and/or counting error. Dishes were placed into our light and temperature-controlled fish room at 25°C. After 2 hours, a 1mL sample is taken from each dish and counted for a post-count of rotifers per mL. Three replicates of individual IVF rounds with the same experimental setup were obtained. Pre and post counts of rotifers per mL were analyzed using a Students T-test, p = 0.05, to assess prey capture success following the 2 hours of exposure. Post counts of each condition of rotifers per mL were taken blind to the experimenter, and power analysis of α = 0.05 and 80% (ClinCalc) was sufficient for each group of statistical testing.

For prey capture on sham, unilateral, or bilateral ablated individuals, *Tg(slc17a6b*:*DsRed)*;*Tg(atoh7*:*eGFP)* larvae that underwent various ablation procedures as noted elsewhere were placed into our light and temperature controlled fish room for 2 hours prior to prey capture to acclimate to room conditions. During this time single drops of rotifers in 15 ppt salt were placed into individual 12 well plates, and a pre-count for each well was obtained (~15–30 rotifers per well). Following pre-count, 15 ppt salt solution was increased to 2 mL final volume, and acclimated fish were then added in 4 mL of 1X E3. Multiple mock wells with no larvae were used to assess rotifer counting. Larvae were then kept in the fish room for 2 hours to perform prey capture, removed from the wells, and then household vinegar was added to each well to immobilize the rotifers, which were then individually counted as they were removed to obtain a post-count. The feeding data for larvae across three rounds of this experiment was binarized for eaters (1) versus non-eaters (0), and a Chi-Square analysis was performed to compare these groups. Mock wells had a counting error of +/- two rotifers, so larvae that consumed more than two rotifers were considered eaters.

For turn bias assays, larvae were raised under standard conditions in E3 as described earlier. At 6 or 7 dpf, larvae from *gsx1*^*y689*^ carrier in-crosses were screened and any with physical abnormalities including under inflated swim bladders were removed. Next, larvae were moved to an environmentally controlled room, maintained at 25°C and constant darkness, for behavior testing and allowed a minimum of 30 minutes adaptation time on a light box at ~70 μW/cm^2^. For experiments, individual larvae were placed into a 10cm dish with E3 media. Four dishes were recorded simultaneously per behavior rig. All recordings were performed under 970nm infrared illumination and captured with a 12mm lens fitted with a long-pass filter. Environmental illumination was provided by a cool white light LED (Thorlabs) adjusted to an intensity of 70 μW/cm^2^. The recording paradigm for each larva was 2.5 minute adaptation to the recording area, 30 sec recording under full field illumination, and 30 sec recording following loss of illumination that was repeated three additional times for a total for four sequential recordings per larva. Recordings were captured at 10 Hz using a μEye IDS1545LM-M CMOS camera. Analysis was performed using custom IDT software as previously described [51,78]. For analysis, we quantified match index (MI), total turn angle (TTA), and fractal dimension. Match index determines the proportion of trials that occur in the same direction as the first trial, quantified as a binary score as 1 for matched and 0 for opposed direction. A value of 1 means trials 2–4 were all in the same direction and 0 that the trials were all in the opposed direction. Total turning is the absolute total of all angular displacement during the recording interval. Fractal dimension provides a measure of the strength of local movement. Values are binned between 1, a path trajectory filling one dimension, and 2, represents a path during the recording interval completely filling a two-dimensional space. For total turning and fractal dimension analysis, the responses over all four trials were averaged for baseline and dark responses. Following behavior testing, all larvae were individually housed and post-hoc genotyped to correlate behavioral performance with genotype.

### Genotyping

Genotyping followed the protocol in [54] for *gsx1*^*y689*^. A small tail portion was dissected from adults, larvae, or embryos to use in DNA extraction. Tails were denatured at 95°C in DNA lysis buffer (10mM Tris pH 7.5, 50mM KCl, 0.3% Tween20, 0.3% Triton X, 1mM EDTA) and digested at 55°C for at least 2h with 10mg/mL proteinase-K (Omega) (1:5 volume dilution). The proteinase-K was heat-inactivated at 95°C before the DNA sample was used in a standard DreamTaq (Thermo Fisher) PCR reaction with gene-specific primers for *gsx1* (Table 1). Amplified fragments were analyzed using 4% agarose gel electrophoresis; *gsx1*^*+/+*^ (wildtype) appear as one larger band (140bp), mutants appear as one smaller band (129bp), and heterozygotes appear as two bands, one at each size.

### RT-PCR for *gsx1* mRNA

Embryos and larvae obtained from TL crosses were raised to the desired ages (30 hpf, 48 hpf, 6 dpf) and stored in RNAlater (Sigma). Total RNA was extracted from 10 dissected eyes and 10 dissected heads without eyes at each age using a phenol chloroform extraction method with TRI-Reagent (Invitrogen). 2μg of cDNA prepared using a 2-step RT-PCR kit (Invitrogen) was used in 28 cycles of PCR with PlatinumTaq (Invitrogen) and intron-spanning gene-specific primers for *gsx1* and *ef1α* (Table 1). Fragments were visualized and imaged using a FluorChemQ imager (ProteinSimple) on a 3% agarose gel with SYBR Safe DNA gel stain (Invitrogen) using a blue light transilluminator (Clare Chemical Research). A Nanodrop fluorospectrometer was used to assess RNA quality before RT-PCR and gel electrophoresis.

### Lipophilic dye injections

7 dpf *Tg(slc17a6b*:*DsRed)* or *Tg(HuC*:*GFP)* larvae were fixed overnight in 4% PFA following 4 days of recovery from unilateral pretectal ablations at 72 hpf with fresh 0.003% PTU in E3 exchanged daily. Larvae were laterally mounted in 1.5% agarose in 1X PBS with the eye contralateral to the ablated side directed surface up. DiI (1mg/mL dissolved in ethanol) (Invitrogen, cat. #D3911) was injected with a WPI Nanoliter 2000 microinjector into temporal-ventral locations within the retina. Samples were incubated for 1 hour at 37°C and then left to diffuse overnight at 4°C. Samples were removed from agarose and mounted dorsally and laterally in 1X PBS. Samples were imaged on a 2-photon or confocal microscope within 24 hours of DiI injection to visualize retinotectal axon patterns.

For retinotectal mapping, *gsx1*^*y689/+*^ adult zebrafish were in-crossed and offspring raised to 6 dpf and fixed with 4% PFA overnight. Larvae were laterally mounted in 1.5% agarose in 1X PBS. DiO (1mg/mL dissolved in DMF) (Invitrogen, cat. # D275) was injected into the nasal retina and samples were left in a 37°C incubator for 1 hour. The same samples were then injected with DiI (1mg/mL dissolved in ethanol) (Invitrogen, cat. #D3911) into temporal-ventral locations within the retina and then left to diffuse overnight at 4°C. Samples were removed from agarose and mounted laterally in 1X PBS and scanned at 2μm depths through the full DiI and DiO labeling on the confocal microscope within 24 hours of injection followed by genotyping.

### Cryostat retinal sections and quantification

6 dpf larvae (*Tg(isl2b*:*GFP);gsx1*^*y689/+*^ in-cross offspring) were fixed in 4% PFA overnight at 4°C. Tissue was prepped by sinking larvae in a 25% sucrose in 1X PBS solution, then 35% sucrose in 1X PBS and mounting in Optical Cutting Temperature (OCT Clear, Fisher HealthCare, 4585). Sections were taken on a Leica CM1850 cryostat at 12μm through the entire retina based on published protocols [95]. Staining procedures for sections followed [95], and anti-GFP antibody and SYTO59 staining were used (Table 1). Images were taken on a laser scanning confocal microscope and analyzed using Vaa3D software to count cells.

### Imaging: Confocal, 2-photon, and epifluorescent microscopy

Confocal images were taken on an Olympus BX61 confocal microscope with Fluoview FV1000 software with imaging objectives 20X, 40X, 60X oil immersion, or 40X silicon oil immersion. Scientifica VivoScope 2-photon microscope with Spectra Physics Mai Tai HP tunable lasers and ScanImage software was used to perform 2-photon laser-mediated ablation experiments, *in vivo* imaging of acridine orange staining, and DiI injections imaging after photo-ablation. Zeiss Axiozoom V16 microscope with fluorescence was used to sort transgene expression and examine antibody labeling following IHC.

### Quantification of RGC axon volumes

Lateral confocal images using *Tg(atoh7*:*eGFP)* and SYTO59 were acquired by imaging through the entire tectal lobe from start of RGC axon fluorescence to end. Images were taken at 2μm per slice and stored as.oib files. Images were converted using Imaris 9.3 file converter and input into Imaris 9.9 (http://www.bitplane.com/imaris/imaris, Oxford Instruments). Masks were taken of the region of interest starting dorsal to AF1 and AF2 labeling to include AF3-10. A mask of RGC axon labeling was then input into Labkit pixel classifier (ImageJ FIJI), and volume rendering was accomplished via a machine learning algorithm. Computed results were sent back to Imaris to obtain a reading of axon volume for our region of interest. Imaris volume rendering and capture was taken blind to the researcher by sample genotype. Volume data was analyzed in IBM SPSS (2021).

### Pretectal ablations

For unilateral pretectal cell ablation to assess RGC axon trajectories and termination, adult fish with transgenes *Tg(slc17a6b*:*DsRed)*;*Tg(atoh7*:*eGFP)* were in-crossed and sorted for both GFP and RFP expression at 48 hpf. 0.003% PTU in 1X E3 was added at 6 hpf to prevent pigmentation and embryos were raised in an incubator at 28.5°C. At 72 hpf embryos were anesthetized with MS-222 and mounted in 1% low-melt agarose in E3 in a 4-well custom glass slide chamber. Each embryo was located using epifluorescence with a 16X objective (Nikon) and then located on the 2-photon at 920nm with 5X magnification to visualization left or right pretectal regions pre-ablation. Scans were taken pre-ablation = detecting *Tg(slc17a6b*:*DsRed)* and *Tg(atoh7*:*eGFP)* at ~15–18μW laser power. Target locations in unilateral pretectal regions were zoomed into using digital zoom features to 65X and laser power was increased to ~94–99μW, exposing the region of *Tg(slc17a6b*:*DsRed)*-positive cells for 1 second. Additional target regions were identified by zooming out and turning down laser power to locate the regions and then zooming back into 65X to expose the second pretectal region of *Tg(slc17a6b*:*DsRed)*-positive cells in the same way. Post-ablation images were taken at 5X zoom to assess ablation success. Displacement of the fluorescent protein can be visualized in post-ablation images to identify successful targeting, and acridine orange was included to visualize dying cells. Larvae were taken out of agarose carefully and recovered in 1X E3 until 0.003% PTU is returned a few hours later. Experiments to assess larval health after unilateral ablations determined that following ablations, 100% of swim bladders inflated and overall health is maintained through 7 dpf. Ablated larvae health was compared to controls that are also mounted and unmounted in 1% agarose in E3 as a sham ablation.

For axon ablation control experiments, adult zebrafish carrying *Tg(atoh7*:*eGFP)* were in-crossed and embryos were collected and placed in 0.003% PTU in E3 with daily exchanges of this rearing solution. At 72 hpf, the right eye of each larva was enucleated, and they were placed immediately in 1X Hanks’ solution (Thermo Fisher) to recover for 2 hours. This allowed for targeting of RGC axon regions on the unilateral side. After recovery, enucleated larvae were mounted in a custom glass slide chamber in 1% agarose in E3 with the enucleated side facing the glass and microscope objective. Larvae were visualized with epifluorescence for precise mounting, and the ventral region of the optic nerve was targeted for ablation. Ablation parameters were the same for this experiment as used previously. Following ablation, larvae are un-mounted and placed back in 0.003% PTU in E3 to recover for 4 days with fresh media exchange and overall health checks performed daily. Control, enucleated larvae are treated the same way as experimental ablated samples except without ablation.

For unilateral and bilateral ablations used for prey capture analyses, adult fish with transgenes *Tg(slc17a6b*:*DsRed)*;*Tg(atoh7*:*eGFP)* were crossed and sorted for both GFP and RFP expression at 48 hpf. 0.003% PTU in 1X E3 was added at 6 hpf to prevent pigmentation and embryos were raised in an incubator at 28.5°C. At 72 hpf zebrafish were mounted in 1% low-melt agarose in E3 in a 6 cm dish. Each embryo was located using epifluorescence and then located on the 2-photon at 920nm with 10x magnification to visualize left or right AF7 regions pre-ablation. Scans were taken pre-ablation detecting *Tg(slc17a6b*:*DsRed)* and *Tg(atoh7*:*eGFP)* at ~35–40% digital Pockel Cell laser power. Target locations in bilateral and right side unilateral AF7 regions were zoomed into using digital zoom features to 70X at the bottom of the AF7 region, and laser power was increased to 100% using the digital laser controller, exposing *Tg(slc17a6b*:*DsRed)*-positive cells twice for 2 seconds each. Following this, the target location was moved halfway into AF7, exposing the *Tg(slc17a6b*:*DsRed)*-positive cells twice for 2 seconds each at 100% laser power. Post-ablation images were taken at 10X to assess ablation success. Displacement of the fluorescent protein can be visualized in post-ablation images to identify successful targeting. Larvae were taken out of agarose carefully and recovered in 1X EVANS physiological solution overnight until replaced with E3 the following morning. E3 was refreshed daily until 7 dpf, when embryos were then assessed for prey capture ability.

### Acridine orange assay

72 hpf *Tg(slc17a6b*:*DsRed)* wildtype zebrafish underwent unilateral pretectal photo-ablation through the aforementioned parameters and were left to recover in E3 for 2 hours. A cell death assay was used to assess photo-ablation success 2 hours post ablation with 5μg/mL of acridine orange (AO) [96] (Sigma) added to 1mL of E3. Ablated larvae (n = 6) and control non-ablated larvae (n = 6) were live mounted in 1% low melt agarose in E3 with 1mL MS-222 added following AO staining and re-imaged on the 2-photon microscope, zoomed into both right and left pretectal regions at a wavelength of 920nm. PMTs detected both AO and *Tg(slc17a6b*:*DsRed)*. Cell death assays were also performed the same way at 6 dpf (3 days post ablation) and 7 dpf (4 days post ablation) to visualize if AO staining was still present after multiple days of recovery. Parameters for larval recovery after ablation and image acquisition were the same for each experiment.

### Image processing, cell counting, and statistical analysis

Images were max projected unless otherwise indicated in Fiji ImageJ with brightness and contrast adjusted to avoid over or under exposure of pixels. Vaa3D (v.3.20) was used to count cells on both pretectum sides. Pretectal cell counts did not indicate significant differences in HuC/D or *Tg(slc17a6b*:*DsRed)* positive cells across right or left sides and therefore were grouped as all representative pretectal lobes (one counted lobe, n = 1). Optic nerve measurements were taken in Fiji ImageJ from max projected z-stack image files and averaged across three individual size measurements for both the right and left optic nerve at the location of the nerve leaving the retina. SPSS one-way ANOVAs and post hoc Tukey’s tests (α = 0.05) were used to determine significant differences between genotypes of pretectal cell counts and optic nerve measurements. Fiji ImageJ Plugin, Z-stack Depth ColorCode 0.0.2, was used to depth code for arborization field visualization and then max projected. Post-ablation depth coded images were analyzed for presence or absence of AF7 formation by examination of coded color for yellow and using blinded analysis of ablated or control lateral view max projection z-stacks. AF9 was matched in depth code to begin with blue coloring as the deepest, most medial AF. Student’s *t* tests were used for statistical comparison of *Tg(slc17a6b*:*DsRed)* counts in ablated versus non-ablated pretecta on the contralateral sides in the same larvae. Outliers for statistical testing were determined by SPSS software for descriptive statistics and confirmed via GraphPad (Grubbs’ test). Image analysis was done blind of the genotype. Power analysis of α = 0.05 and 80% (ClinCalc) was sufficient for each group used in statistical testing.

## Supporting information

S1 Fig *Tg(dlx5/6a*:*GFP)* is a marker for inhibitory neuron connections to the pretectum that are missing in *gsx1*^*y689*^. **(A-B)** Max projection of confocal z-stacks through pretectal region (~20μm), in **(A-A**^**iii**^**)** *gsx1*^*+/+*^ and **(B-B**^**iii**^**)** *gsx1*^*y689*^. HuC/D = blue, *Tg(dlx5/6a*:*GFP)* = green, *Tg(slc17a6b*:*DsRed)* = magenta. White dashed line outlines pretectal region. Cyan line indicates arborization field (AF7) region with inhibitory connections to this region that appear to be missing in *gsx1* mutants compared to *gsx1*^*+/+*^.
(TIF)

S2 Fig *Tg(slc17a6b)*-positive neurons surround AF7. **(A-A**^**iii**^**)** Dorsal view of partial projection of confocal z-stacks (~5μm). **(B-B**^**iii**^**)** Lateral view partial projection (~5μm). *Tg(atoh7*:*eGFP)* = RGC axons (green), anti-Znp1 = presynaptic terminals (magenta), *Tg(slc17a6b*:*DsRed)* = glutamatergic neurons (blue), merge = showing AF7 location (green and magenta area). Yellow dashed line outlines Pr region, white dotted line outlines AF7 neuropil. Schematics of orientation are in (A, B).
(TIF)

S3 Fig *gsx1* is not expressed in the eye or required for retinal cell type specification or morphology. **(A-A**^**ii**^**)** RT-PCR confirming *gsx1* is not expressed in the eye at **(A)** 30 hpf, **(A**^**i**^**)** 48 hpf, **(A**^**ii**^**)** 6 dpf. 3% agarose gel, white arrow indicates ~800bp *gsx1* fragment. 1 = eye cDNA, 2 = head cDNA. *ef1α* used as a control for DNA quality. ^ = 100bp ladder. Cyan arrowhead = 500bp marker. **(B-D)** Max projections of confocal z-stacks (12μm) of retinal sections at 6 dpf in **(B)** wildtype, **(C)** *gsx1*^*y689/+*^, **(D)** *gsx1*^*y689*^. Green = *Tg(isl2b*:*GFP)*, labeling retinal ganglion cells (RGC) in the ganglion cell layer (GCL). Blue = SYTO59, labeling nuclei. White arrow indicates optic nerve leaving the GCL and white dashed outline indicates GCL that is quantified. Scalebar = 100μm. **(E)** Box and whisker plot of GCL quantification for positive RGCs per individual eyes, average taken across 3 consecutive sections with the optic nerve present. One-way ANOVA resulted in no significant differences found across genotypes, *gsx1*^*+/+*^ (n = 16), *gsx1*^*y689/+*^ (n = 9), *gsx1*^*y689*^ (n = 19), F(2,41) = 0.11, *p* = 0.90.
(TIF)

S4 Fig Optic chiasm crossing and optic nerve width are normal in *gsx1* mutants. **(A-C)** Max projection of confocal z-stacks (~55μm) in *Tg(isl2b*:*GFP)* (green, RGCs) showing normal optic chiasm (white arrowhead) and optic nerve (ON) width at 48 hpf in, **(A)** *gsx1*^*+/+*^ (n = 33), **(B)** *gsx1*^*y689/+*^ (n = 21), and **(C)** *gsx1*^*y689*^ (n = 36). Bracket outlines optic nerve leaving the eye where measurements were taken. **(D)** Box and whisker plot of measurements for both the right and left ON for each genotype with no statistical differences found, single factor ANOVA [F(2,59) = 0.76, *p* = 0.47].
(TIF)

S5 Fig The optic nerve regenerates over the course of four days post 2-photon laser induced injury. **(A)** Experimental timeline and region of targeted ablation following enucleation at 72 hpf. **(B-C)** Lateral orientation of pre and post ablation of max projected 2P z-stacks (~80μm). Red boxes show where ablation took place. Post image red arrows indicate displacement of fluorescent protein following ablation of RGC axons in *Tg(atoh7*:*eGFP)*. Scalebar = 100μm. **(D-E)** 7 dpf max projections of confocal z-stacks in *Tg(atoh7*:*eGFP)* in **(D)** control non-ablated (n = 7) and **(E)** 72 hpf ablated ventral optic nerve (n = 8), (~90μm). **(Di, Ei)** RGC axons are depth color coded to provide reference for certain AFs, such as the white arrowhead indicating regeneration of AF6 (yellow). Scalebar = 100μm.
(TIF)

S1 Raw Data Numerical data that underlies graphs or summary statistics. (XLSX)
