# Supplementary figures and images for: Transcriptional control of visual neural circuit development by GS homeobox 1
Source: PLoS Genet. 2024 Apr 26;20(4):e1011139. doi: 10.1371/journal.pgen.1011139 (PMC11051655; doi:10.1371/journal.pgen.1011139)

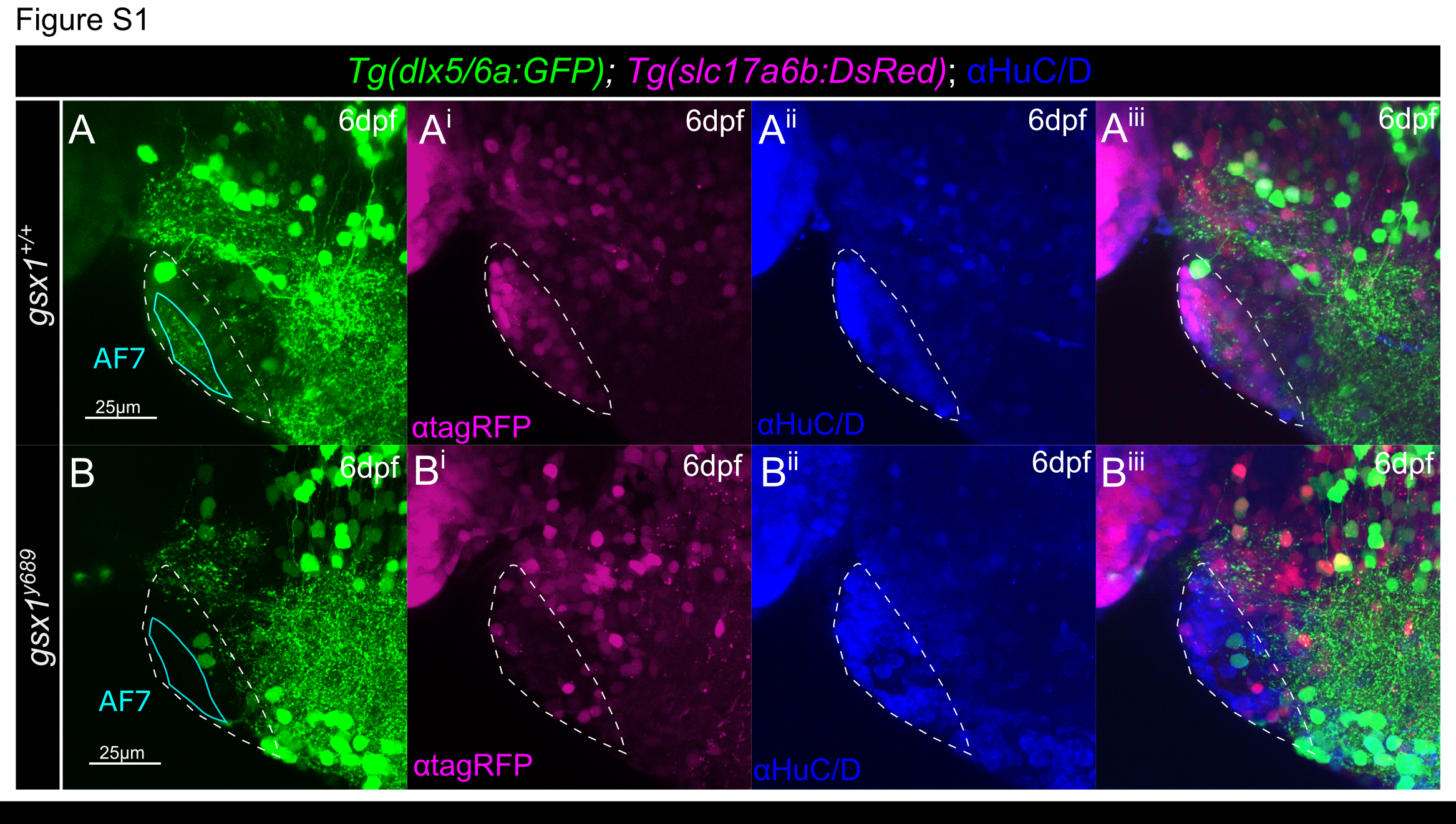

Supplement: S1 Fig — (A-B) Max projection of confocal z-stacks through pretectal region (~20μm), in (A-Aiii) gsx1+/+ and (B-Biii) gsx1y689. HuC/D = blue, Tg(dlx5/6a:GFP) = green, Tg(slc17a6b:DsRed) = magenta. White dashed line outlines pretectal region. Cyan line indicates arborization field (AF7) region with inhibitory connections to this region that appear to be missing in gsx1 mutants compared to gsx1+/+. (TIF) [file pgen.1011139.s001.tif]

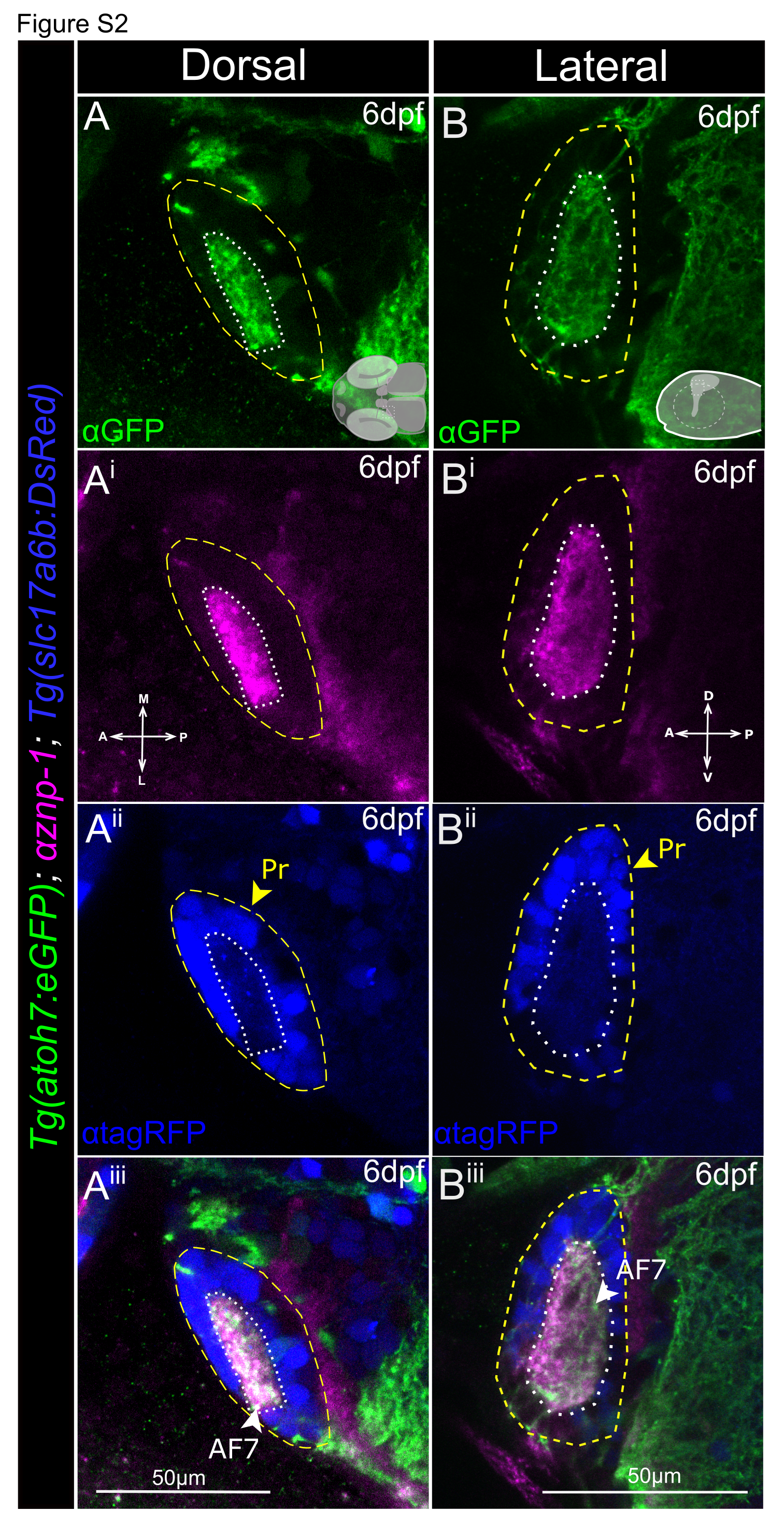

Supplement: S2 Fig — (A-Aiii) Dorsal view of partial projection of confocal z-stacks (~5μm). (B-Biii) Lateral view partial projection (~5μm). Tg(atoh7:eGFP) = RGC axons (green), anti-Znp1 = presynaptic terminals (magenta), Tg(slc17a6b:DsRed) = glutamatergic neurons (blue), merge = showing AF7 location (green and magenta area). Yellow dashed line outlines Pr region, white dotted line outlines AF7 neuropil. Schematics of orientation are in (A, B). (TIF) [file pgen.1011139.s002.tif]

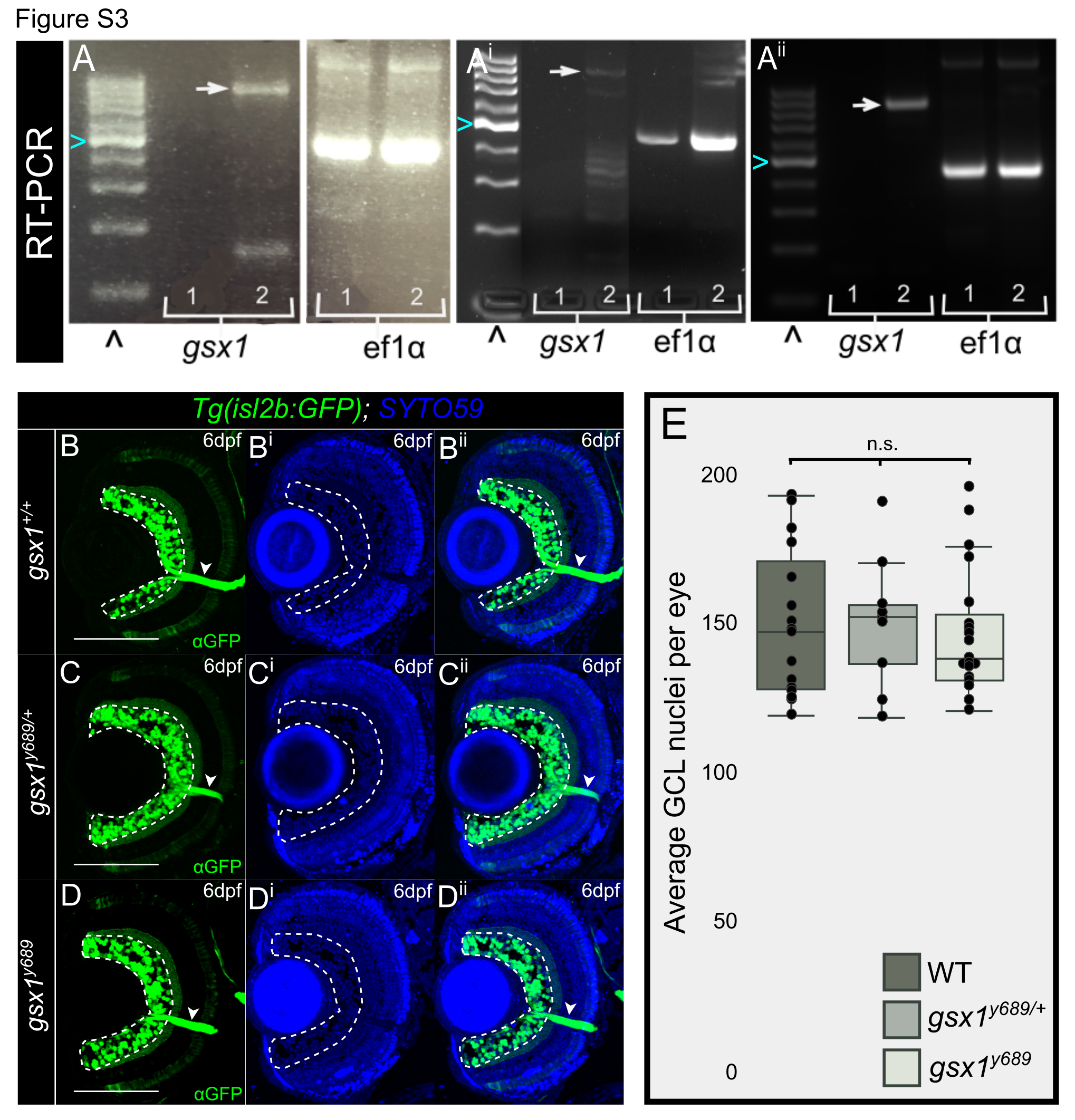

Supplement: S3 Fig — (A-Aii) RT-PCR confirming gsx1 is not expressed in the eye at (A) 30 hpf, (Ai) 48 hpf, (Aii) 6 dpf. 3% agarose gel, white arrow indicates ~800bp gsx1 fragment. 1 = eye cDNA, 2 = head cDNA. ef1α used as a control for DNA quality. ^ = 100bp ladder. Cyan arrowhead = 500bp marker. (B-D) Max projections of confocal z-stacks (12μm) of retinal sections at 6 dpf in (B) wildtype, (C) gsx1y689/+, (D) gsx1y689. Green = Tg(isl2b:GFP), labeling retinal ganglion cells (RGC) in the ganglion cell layer (GCL). Blue = SYTO59, labeling nuclei. White arrow indicates optic nerve leaving the GCL and white dashed outline indicates GCL that is quantified. Scalebar = 100μm. (E) Box and whisker plot of GCL quantification for positive RGCs per individual eyes, average taken across 3 consecutive sections with the optic nerve present. One-way ANOVA resulted in no significant differences found across genotypes, gsx1+/+ (n = 16), gsx1y689/+ (n = 9), gsx1y689 (n = 19), F(2,41) = 0.11, p = 0.90. (TIF) [file pgen.1011139.s003.tif]

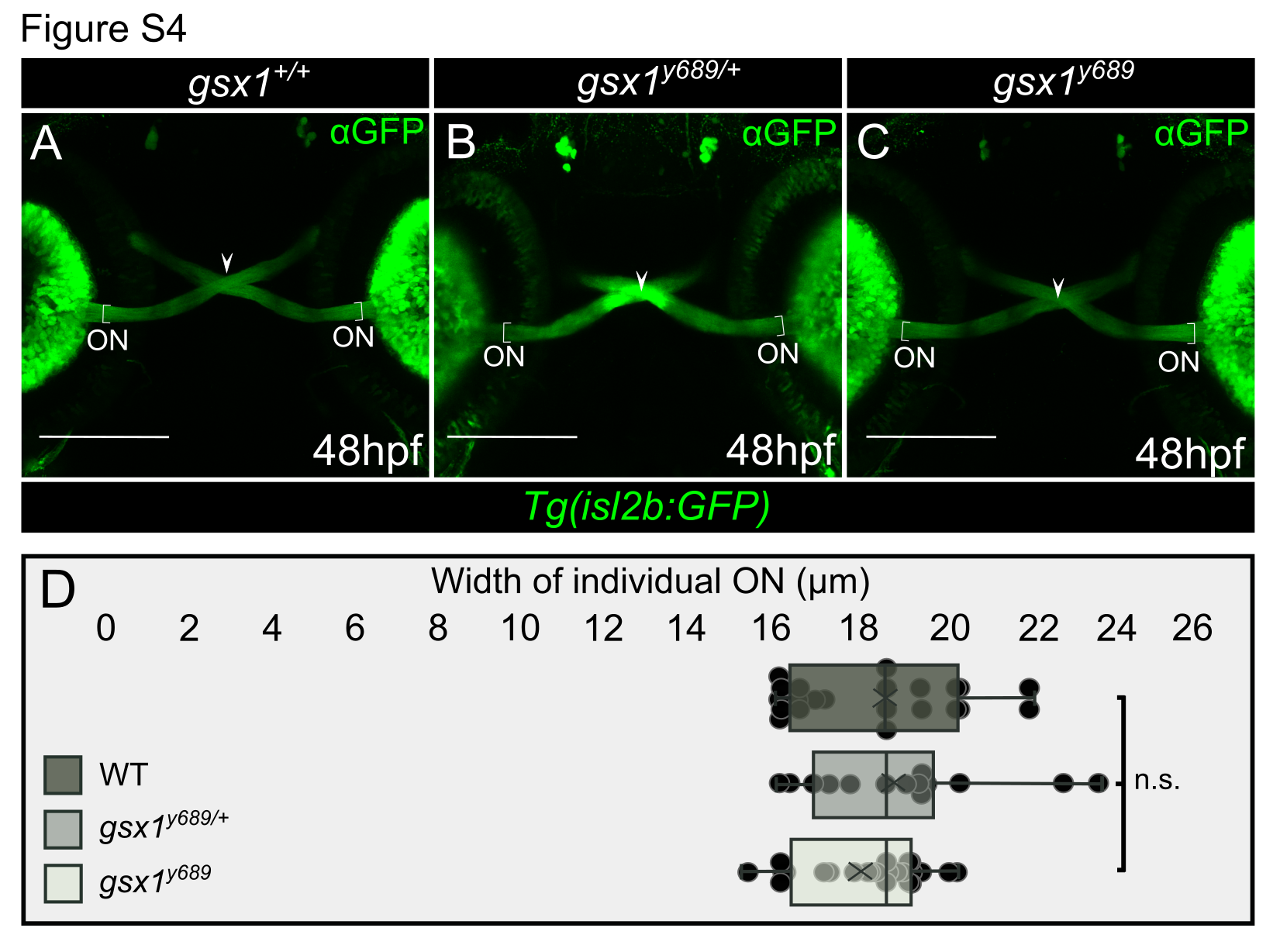

Supplement: S4 Fig — (A-C) Max projection of confocal z-stacks (~55μm) in Tg(isl2b:GFP) (green, RGCs) showing normal optic chiasm (white arrowhead) and optic nerve (ON) width at 48 hpf in, (A) gsx1+/+ (n = 33), (B) gsx1y689/+ (n = 21), and (C) gsx1y689 (n = 36). Bracket outlines optic nerve leaving the eye where measurements were taken. (D) Box and whisker plot of measurements for both the right and left ON for each genotype with no statistical differences found, single factor ANOVA [F(2,59) = 0.76, p = 0.47]. (TIF) [file pgen.1011139.s004.tif]

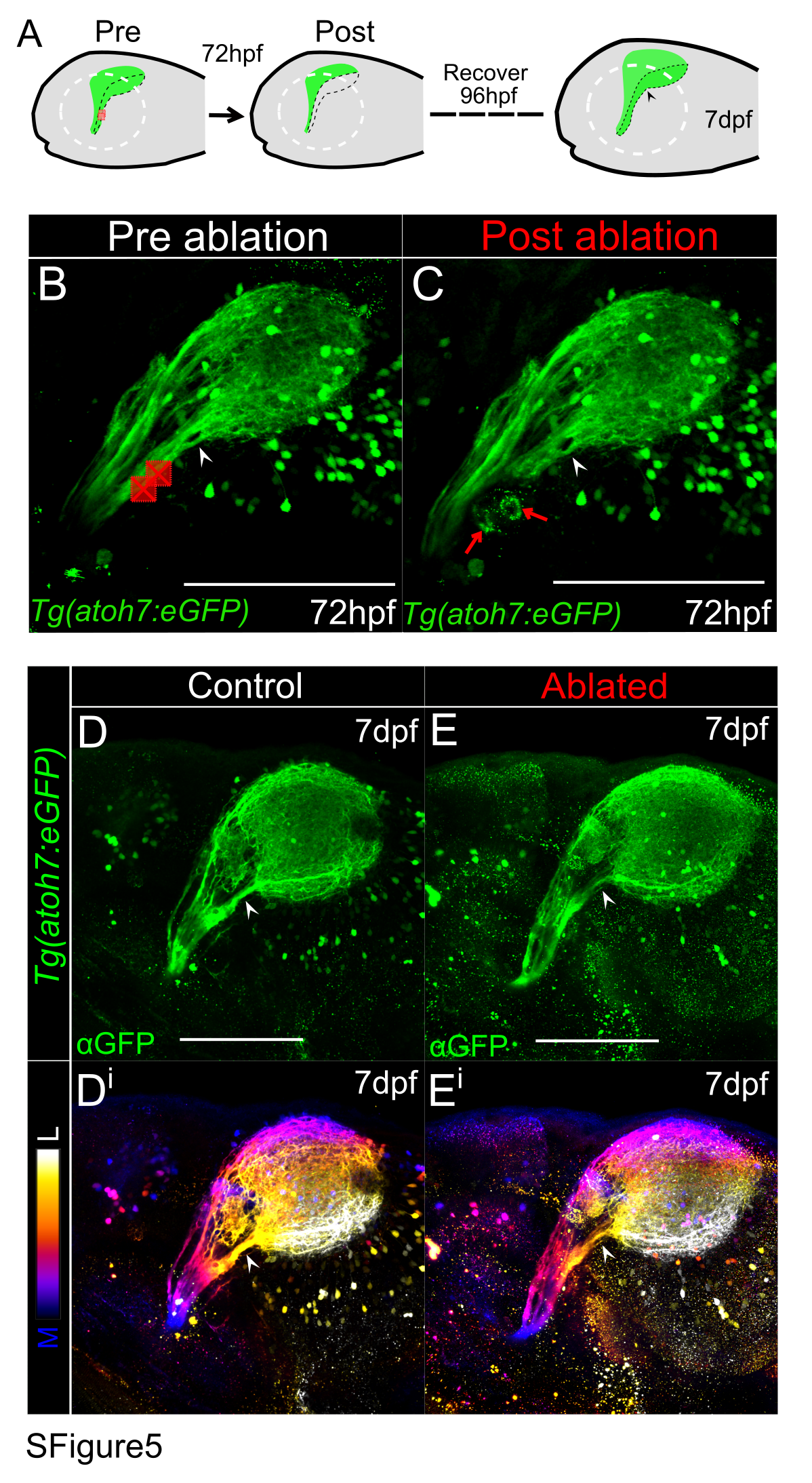

Supplement: S5 Fig — (A) Experimental timeline and region of targeted ablation following enucleation at 72 hpf. (B-C) Lateral orientation of pre and post ablation of max projected 2P z-stacks (~80μm). Red boxes show where ablation took place. Post image red arrows indicate displacement of fluorescent protein following ablation of RGC axons in Tg(atoh7:eGFP). Scalebar = 100μm. (D-E) 7 dpf max projections of confocal z-stacks in Tg(atoh7:eGFP) in (D) control non-ablated (n = 7) and (E) 72 hpf ablated ventral optic nerve (n = 8), (~90μm). (Di, Ei) RGC axons are depth color coded to provide reference for certain AFs, such as the white arrowhead indicating regeneration of AF6 (yellow). Scalebar = 100μm. (TIF) [file pgen.1011139.s005.tif]
